# Lung endothelium, tau, and amyloids in health and disease

**DOI:** 10.1152/physrev.00006.2023

**Published:** 2023-08-10

**Authors:** Ron Balczon, Mike T. Lin, Sarah Voth, Amy R. Nelson, Jonas C. Schupp, Brant M. Wagener, Jean-Francois Pittet, Troy Stevens

**Affiliations:** ^1^Department of Biochemistry and Molecular Biology, University of South Alabama, Mobile, Alabama, United States; ^2^Department of Physiology and Cell Biology, University of South Alabama, Mobile, Alabama, United States; ^3^Department of Internal Medicine, University of South Alabama, Mobile, Alabama, United States; ^4^Center for Lung Biology, University of South Alabama, Mobile, Alabama, United States; ^5^Department of Cell Biology and Physiology, Edward Via College of Osteopathic Medicine, Monroe, Louisiana, United States; ^6^Pulmonary and Critical Care Medicine, Department of Internal Medicine, Yale University, New Haven, Connecticut, United States; ^7^Department of Respiratory Medicine, Hannover Medical School, Hannover, Germany; ^8^German Center for Lung Research (DZL), Hannover, Germany; ^9^Department of Anesthesiology and Perioperative Medicine, University of Alabama-Birmingham, Birmingham, Alabama, United States

**Keywords:** acute respiratory distress syndrome, beta-amyloid (Aβ), infection, pneumonia, tauopathy

## Abstract

Lung endothelia in the arteries, capillaries, and veins are heterogeneous in structure and function. Lung capillaries in particular represent a unique vascular niche, with a thin yet highly restrictive alveolar-capillary barrier that optimizes gas exchange. Capillary endothelium surveys the blood while simultaneously interpreting cues initiated within the alveolus and communicated via immediately adjacent type I and type II epithelial cells, fibroblasts, and pericytes. This cell-cell communication is necessary to coordinate the immune response to lower respiratory tract infection. Recent discoveries identify an important role for the microtubule-associated protein tau that is expressed in lung capillary endothelia in the host-pathogen interaction. This endothelial tau stabilizes microtubules necessary for barrier integrity, yet infection drives production of cytotoxic tau variants that are released into the airways and circulation, where they contribute to end-organ dysfunction. Similarly, beta-amyloid is produced during infection. Beta-amyloid has antimicrobial activity, but during infection it can acquire cytotoxic activity that is deleterious to the host. The production and function of these cytotoxic tau and amyloid variants are the subject of this review. Lung-derived cytotoxic tau and amyloid variants are a recently discovered mechanism of end-organ dysfunction, including neurocognitive dysfunction, during and in the aftermath of infection.

## Graphical abstract

**Figure:**
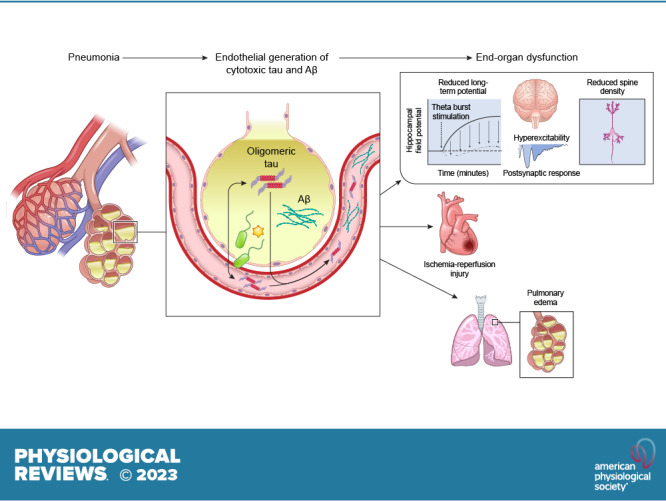

CLINICAL HIGHLIGHTS The acute respiratory distress syndrome is characterized by pulmonary edema and refractory hypoxemia. Infection is a common cause of the acute respiratory distress syndrome. Infection-induced acute respiratory distress syndrome leads to end-organ dysfunction, with remarkably high rates of morbidity and mortality among surviving patients, even years after their critical illness. Infection elicits the production of cytotoxic tau and beta-amyloid variants within the lung. Cytotoxic tau and beta-amyloid generated within the lung contribute to lung injury during infection, and they can disseminate through the circulation to peripheral organs, where they can contribute to end-organ dysfunction, including neurocognitive dysfunction.

## 1. INTRODUCTION

**FIGURE 1.**
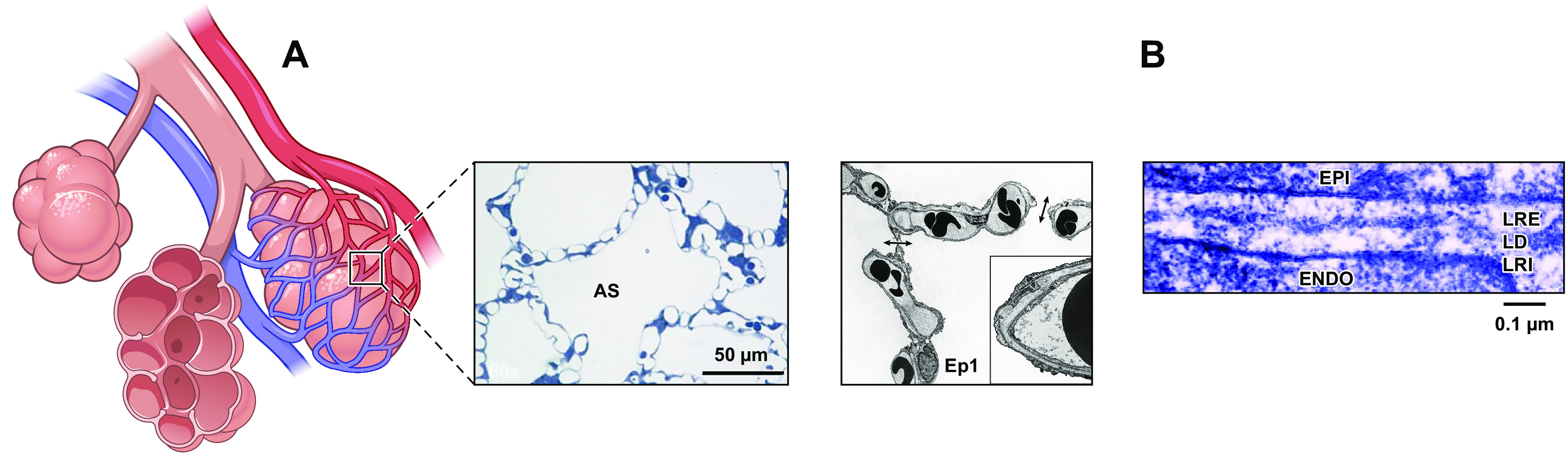
The alveolar-capillary membrane is comprised of type I and type II epithelial cells in close apposition to capillary endothelial cells. *A*: the thin section of the alveolar-capillary barrier is formed by type I cells connected to capillary endothelial cells via a fused basement membrane, which optimizes gas exchange. The dense capillary network perfusing alveoli is illustrated by the light (*center*) and transmission electron (*right*) microscopy images. AS denotes alveolar space (adapted from Ref. 3, with permission from *Journal of Surgical Research*). Ep1 designates a type I epithelial cell, and the arrows illustrate alveolar pores (adapted from Ref. 4, with permission from *American Journal of Respiratory and Critical Care Medicine*). *B*: a high-power electron micrograph illustrates the fused basement membrane separating the capillary endothelium (ENDO) and the type I epithelium (EPI) in the alveolar-capillary network. The basement membrane is comprised of lamina rara externa (LRE), lamina densa (LD), and lamina rara interna (LRA) (Adapted from Refs. 5, 6, with permission from American Physiological Society and *Journal of Cell Biology*, respectively).

The alveolar-capillary membrane is a highly specialized structure, optimized to facilitate efficient gas exchange within the lung (1, 2). The gas exchange barrier comprises type I epithelial cells that are immediately juxtaposed to capillary endothelial cells, connected via a fused basement membrane (FIGURE 1). Under normal circumstances, alveolar ventilation is nearly perfectly matched with blood flow across the lung (7–17). Lower respiratory tract infection, as in pneumonia, is a pathophysiological challenge to the alveolar-capillary membrane. The host senses and responds to this challenge by activating both innate and adaptive immune systems (18–24). The alveolar-capillary membrane becomes disrupted, oftentimes leading to development of the acute respiratory distress syndrome, with concomitant alveolar edema and refractory hypoxemia. Whereas pneumonia and the acute respiratory distress syndrome have historically been considered acute processes, it has become evident that they have long-term consequences (20, 25). Morbidity and mortality rates among survivors are extremely high, because of insidious end-organ dysfunction, yet the mechanisms responsible for protracted end-organ dysfunction are unknown. In recent years investigators have discovered that microorganisms utilize their virulence arsenal to communicate with cells within the alveolar-capillary membrane, including capillary endothelial cells, to promote the generation of cytotoxic variants of tau and beta-amyloid (Aβ) (26–34). These cytotoxic variants are heat-stable, protease-resistant, transmissible, self-replicating proteins. They have been recovered in the bronchoalveolar lavage fluid, circulation, and cerebrospinal fluid of patients with ongoing infection, where they contribute to end-organ dysfunction. This review highlights *1*) endothelial phenotypes within the alveolar-capillary membrane; *2*) expression of tau and amyloid precursor protein in lung capillary endothelium; *3*) mechanisms microorganisms utilize to elicit production of cytotoxic tau and Aβ variants; and *4*) the consequences of generation of cytotoxic tau and Aβ in the lung, circulation, heart, and brain.

## 2. THE PULMONARY CIRCULATION AND LUNG ENDOTHELIUM

### 2.1. The Pulmonary Circulation

The pulmonary circulation accommodates 100% of the cardiac output while maintaining low pulmonary vascular pressures (i.e., ∼25/8 mmHg). Blood returning from the systemic circulation enters the pulmonary circulation via the right ventricle, and it is then distributed through the pulmonary arteries and arterioles into the capillaries, where gas exchange occurs. Blood perfused through the pulmonary circulation is deoxygenated (i.e., mixed venous); its blood gas composition reflects the average metabolic activity of all systemic circulations. Therefore, blood coming into the lung’s microcirculation usually has basal arterial partial pressure of oxygen ($Pa_{O_{2}}$) and carbon dioxide ($Pa_{CO_{2}}$) and pH values of approximately 40 mmHg, 46 mmHg, and 7.3–7.35, respectively, at sea level (P_BAR_ ∼ 760 mmHg). Gas exchange begins as blood enters the small precapillary and capillary microvessels that perfuse through respiratory bronchioles, alveolar ducts, and the alveoli. The principal site of gas exchange is the alveolar-capillary membrane, which is anatomically designed to optimize gas exchange efficiency with an extremely thin blood-gas barrier (1, 2). A single red blood cell that is perfused through the lung’s capillary plexus will contact multiple alveolar units (35–39). It is in contact with the gas exchange barrier for up to 1 s at resting cardiac outputs and pressures. The red blood cell offloads CO_2_ and onloads O_2_ within a quarter of a second, illustrating the lung’s capacity to ensure adequate oxygenation. Blood gases (both CO_2_ and O_2_) are precisely controlled within the physiological range. The newly oxygenated blood is returned via pulmonary veins to the left atrium and then the left ventricle for distribution to the systemic circulation.

### 2.2. Lung Endothelial Cell Heterogeneity: from Arteries to Capillaries to Veins

Endothelium is in intimate contact with blood perfusing through the pulmonary circulation. Endothelial cells can be characterized by their morphological appearance (40, 41). They are continuous (i.e., reside on a basement membrane, in intimate contact with neighboring cells through junctional proteins, as in the heart, brain, and muscle), discontinuous (i.e., lack a basement membrane and exhibit large openings between endothelial cells, as in the liver sinusoids), or fenestrated (i.e., reside on a basement membrane and possess transcellular openings, as in the kidney glomerulus). In the lung, the endothelium is of the continuous subtype. However, within the lung the endothelial cell phenotype varies remarkably along the vascular axis, from the arterial to the capillary to the venous segments of the circulation (42–45).

**FIGURE 2.**
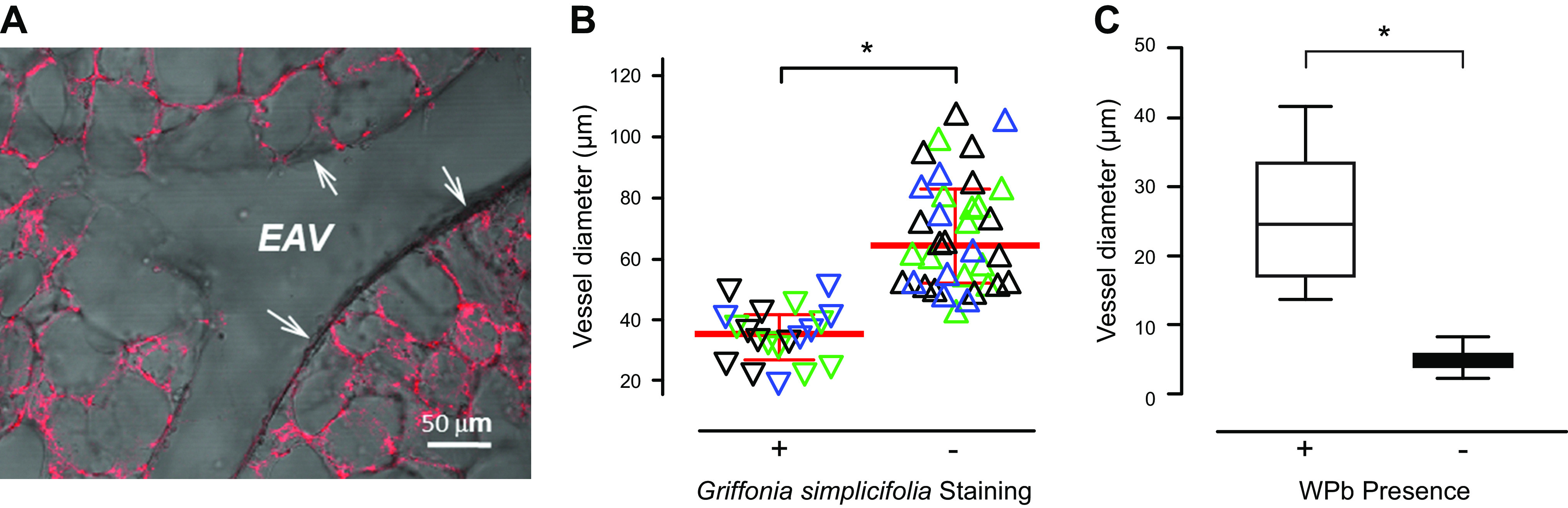
Pulmonary microvascular endothelial cells can be discriminated from pulmonary artery endothelial cells based on lectin binding criteria and the presence and absence of Weibel–Palade bodies (WPb). *A*: lung capillary endothelial cells interact with *Griffonia simplicifolia* lectin, as shown by the red fluorescence in this lung section. EAV denotes an extra-alveolar vessel that does not interact with *G. simplicifolia* (arrows). *B*: quantitation of the vessel diameters reveals that endothelial cells lining blood vessels smaller than 38 µm uniformly interact, whereas endothelial cells lining blood vessels larger than 60 µm do not interact, with *G. simplicifolia*. Endothelial cells lining blood vessels within the 38- to 60-µm range may or may not interact with *Griffonia* lectin, indicating a site in which the cellular transition occurs. *C*: Weibel–Palade bodies are found in pulmonary artery endothelial cells, but they are not found in the capillary segments of the normotensive pulmonary circulation. *Significant difference (*P* < 0.05). Adapted from Ref. 48, with permission from *Pulmonary Circulation*.

A majority of the work resolving segment-specific endothelial cell specification has focused on the arterial-to-capillary transition. The anatomical variability in these cell types is visible in histological and ultrastructural studies, where features like the cellular dimensions (width by height by length), the number and nature of cell-to-cell contacts, the nature of the basement membrane, and the organelle distribution have been quantified (46, 47). Lectin binding has been useful in further resolving lung endothelial cell phenotypes (FIGURE 2). Whereas *Helix pomatia* (α-GalNAc nominal specificity) recognizes endothelial cells in the arterial segment, *Griffonia simplicifolia* (α-galactose nominal specificity) recognizes endothelial cells in the capillary segment (47, 48). The physiological function of these cell types is different. Capillary endothelial cells form a more restrictive barrier to water, solutes, and macromolecules than do pulmonary artery endothelial cells (42, 46, 49–52). They reside on a fused basement membrane and are immediately adjacent to alveolar epithelial cells; the basement membrane contributes to integrity of the alveolar-capillary membrane and is a source of heparan sulfate glycosaminoglycans, matrikines, and proangiogenic factors (5, 6, 53–56). The capillary endothelial cell barrier is supported by restrictive adherens and tight junctions and by interaction of focal adhesions with the underlying matrix, held under tension via a cortical actin rim, as highlighted in detailed reviews (57–61). The enhanced barrier property of capillary endothelium has been described as the most significant safety factor that protects against alveolar flooding (62). Each cell type expresses a unique complement of ion channels that are linked to site-specific physiological function. For example, the activation of store-operated calcium entry channels is sufficient to increase permeability in the extra-alveolar arteries and veins (43, 46, 63, 64), whereas activation of the mechano-sensitive vanilloid channel of the transient receptor potential proteins (i.e., TRPV4) is sufficient to increase capillary permeability (3, 65–71). T-type voltage-gated calcium channels are expressed in capillaries, and their activation increases P-selectin surface expression and neutrophil recruitment but does not increase permeability (72–74). Discrete endothelial cell phenotypes are evident from anatomical, physiological, and molecular studies, revealing the highly specialized nature of arterial versus capillary endothelium.

It is notable that these discrete cellular features, especially lectin binding, can be selected for during cell isolations, enabling expansion of cell populations that retain physiological features of the in vivo specialization. For example, enriching for cell populations that are *Griffonia simplicifolia* positive, along with other endothelial cell markers like CD31, selects for cells with a capillary phenotype (42). Just as in in vivo studies, these cells exhibit a more restrictive barrier to water, solutes, and proteins compared with pulmonary artery endothelial cells (49). Activation of store-operated calcium entry does not, yet activation of TRPV4 does, increase capillary endothelial cell permeability. Activation of T-type calcium channels leads to P-selectin surface expression and neutrophil transmigration. These cells are also hyperproliferative and exhibit an exquisite single-cell replication competency, suggesting that the lung’s capillary niche is enriched with progenitor cells (75–77). Endothelial cells in general are glycolytic in nature, yet capillary endothelial cells highly depend upon aerobic glycolysis to meet their metabolic requirements (75, 76, 78). Capillary endothelia exhibit an increased acid tolerance along with their increased lactate production (79, 80). Studies utilizing these well-characterized approaches to enrich for endothelial cells with either pulmonary artery or capillary specification improve translation between in vitro and in vivo results.

Studies have been undertaken to resolve where in the circulation the transition occurs between a pulmonary artery and a capillary endothelial cell type. With *Griffonia simplicifolia* as a guide, precapillary arterioles with an inner diameter of ∼38–60 µm were identified as the key transition zone (FIGURE 2) (48). A lung slice model was used to address this issue. Gelatin was infused into the circulation, and agarose was introduced into the airways with physiological pressures. Approximately 300-µm sections were cut and incubated with *G. simplicifolia*. The size of blood vessels that were positive for lectin binding was analyzed. In this study, endothelium of all blood vessels with internal diameters < 38 µm was *Griffonia* positive and endothelium of all blood vessels with internal diameters > 60 µm was *Griffonia* negative. Endothelium in blood vessels ranging between 38 and 60 µm could be either *Griffonia* positive or negative, indicating an essential site where the phenotypic transition occurs.

Endothelial specification was further revealed by studies analyzing the distribution of Weibel–Palade bodies (48, 81–83). Weibel–Palade bodies are subcellular organelles that store von Willebrand factor and release it upon stimulation during inflammation as a mechanism driving platelet adhesion to sites of vascular injury. Multiple other inflammatory proteins are stored in and released with Weibel–Palade bodies, serving to promote hemostasis, inflammation, and angiogenesis (84–91). However, not all lung endothelial cells possess Weibel–Palade bodies. Ultrastructural analysis found that in the normotensive circulation endothelial cells in blood vessels < 20 µm do not possess the organelle, suggesting that the capillary endothelial cells do not participate in the stimulated secretion of von Willebrand factor (48, 81–83). Yet this also means that within blood vessels ranging in size from 20 to 38 µm capillary-like endothelial cells in precapillary vessels can possess Weibel–Palade bodies. Small precapillary arterioles represent an important transition zone of high physiological significance.

Emergence of single-cell RNA sequencing (scRNAseq) approaches has added clarity to the molecular diversity of endothelial cell populations within the lung (92–102). To perform scRNAseq, lung tissue was digested into single-cell populations with proteolytic enzymes and mRNA profiled, revealing molecular archetypes typical of pulmonary artery, capillary, and venous specification. Analyses of multiple different datasets were recently compiled into a lung endothelial cell atlas for human and mouse (99). Endothelial cell populations could be discriminated from other lung cells, where 147 genes were specific to all endothelia within the lung. Lung vascular endothelial cells could be discriminated from lymphatic endothelial cells, where 142 genes were discriminating between these cell types and were common among vascular endothelia. Separation of these cell populations based upon their molecular characterization enabled unbiased assessment of the endothelial archetypes.

**FIGURE 3.**
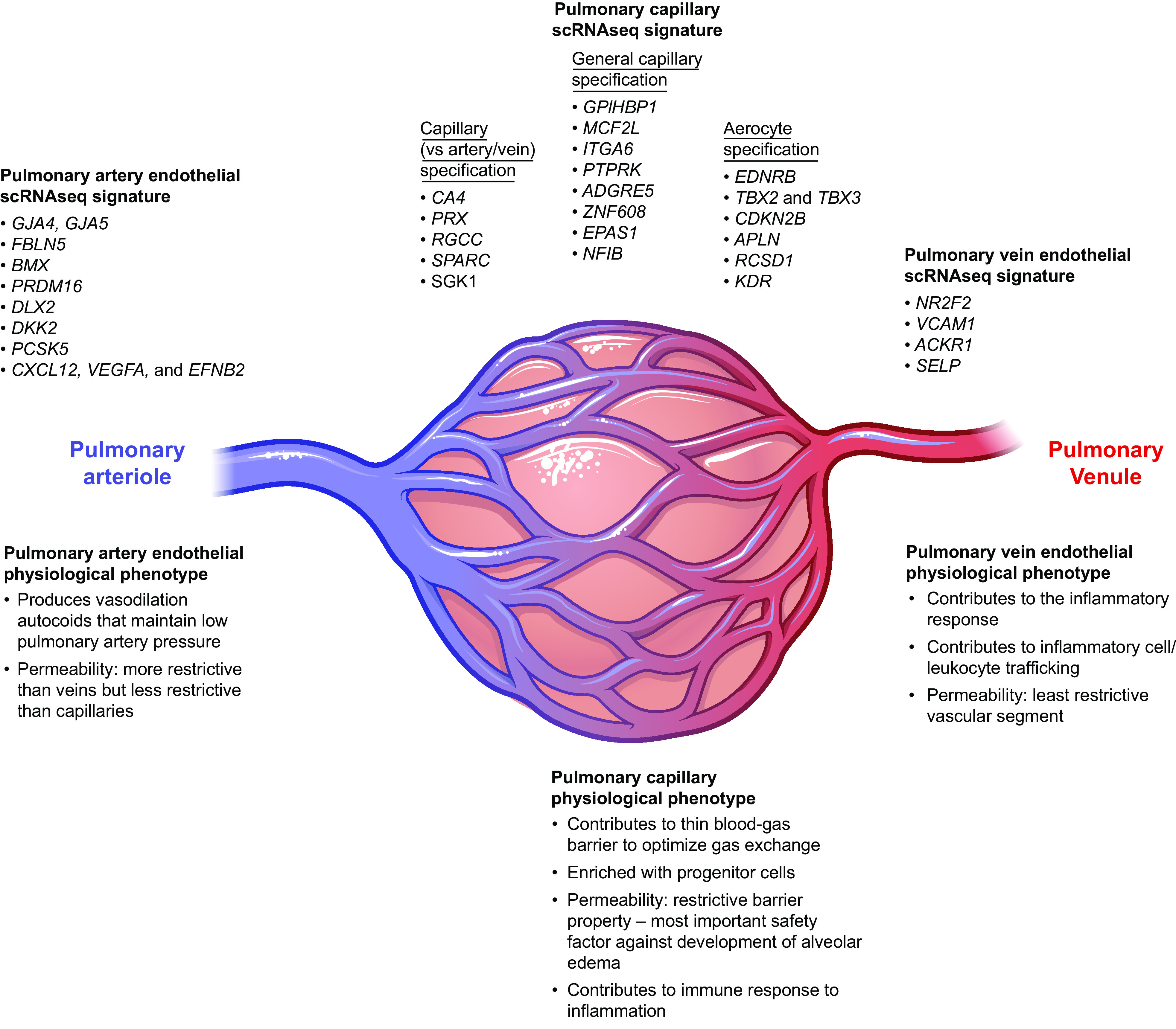
Summary of the gene expression pattern and physiological functions unique to the arterial, capillary, and vein pulmonary endothelial cell phenotypes. Blue represents mixed venous blood gas, and red represents oxygenated blood gas. The pulmonary artery signature is characterized by expression of Gap Junction Protein Alpha 4 (*GJA4*), Gap Junction Protein Alpha 5 (*GJA5*), Fibulin 5 (*FBLN5*), BMX Non-Receptor Tyrosine Kinase (*BMX*), PR/SET Domain 16 (*PRDM16*), Distal-Less Homeobox 2 (*DLX2*), Dickkopf WNT Signaling Pathway Inhibitor 2 (*DKK2*), Proprotein Convertase Subtilisin/Kexin Type 5 (*PCSK5*), C-X-C Motif Chemokine Ligand 12 (*CXCL12*), Vascular endothelial cell growth factor A (*VEGFA*), and Ephrin B2 (*EFNB2*). The capillary signature is characterized by expression of Carbonic Anhydrase 4 (*CA4*), Periaxin (*PRX*), Regulator of Cell Cycle (*RGCC*), Secreted Protein Acidic and Cysteine Rich (*SPARC*), and Serum/Glucocorticoid Regulated Kinase 1 (*SGK1*). The general capillary endothelial cell signature is characterized by expression of Glycosylphosphatidylinositol Anchored High Density Lipoprotein Binding Protein 1 (*GPIHBP1*), MCF.2 Cell Line Derived Transforming Sequence Like (*MCF2L*), Integrin Subunit Alpha 6 (*ITGA6*), Protein Tyrosine Phosphatase Receptor Type K (*PTPRK*), Adhesion G Protein-Coupled Receptor E5 (*ADGRE5*), Zinc Finger Protein 608 (*ZNF608*), Endothelial PAS Domain Protein 1 (*EPAS1*), and Nuclear Factor I B (*NFIB*). The aerocyte signature is characterized by expression of Endothelin Receptor Type B (*EDNRB*), T-Box Transcription Factors 2 and 3 (*TBX2* and *TBX3*), Cyclin Dependent Kinase Inhibitor 2B (*CDKN2B*), Apelin (*APLN*), RCSD Domain Containing 1 (*RCSD1*), and Kinase Insert Domain Receptor (*KDR*). The vein endothelial cell signature is characterized by expression of Nuclear Receptor Subfamily 2 Group F Member 2 (*NR2F2*), Vascular Cell Adhesion Molecule 1 (*VCAM1*), Atypical Chemokine Receptor 1 (Duffy Blood Group) (*ACKR1*), and Selectin P (*SELP*). scRNAseq, single-cell RNA sequencing. Figure created with BioRender.com, with permission.

The pulmonary arterial endothelial cell phenotype was characterized by the gap junction proteins/connexins (GJA4, GJA5) and other structural proteins such as fibulin 2 (FBLN5), Wnt inhibitor (DLL2), Notch ligand (DKK2), tyrosine kinase (BMX), transcription factor (PRDM16), convertase (PCSK5), and cytokines (CXCL12, VEGFA, and EFNB2) (99). STRING interaction networks reveal that these genes encode for pathways central to the function of conduit endothelium, including the elastic properties of blood vessels, communication to adjacent cells through gap junctions and several cytokines, vascular guidance and ephrin- and notch-mediated signaling, and angiogenesis. It is notable that this characterization is representative of physiological features of pulmonary artery endothelium in vivo (FIGURE 3).

A capillary endothelial cell phenotype was resolved by the expression of five genes, including *CA4* (Carbonic anhydrase 4), *PRX* (periaxin), *RGCC* (regulator of cell cycle), *SPARC* (Secreted protein acidic and cysteine rich), and *SGK1* (Serum/glucocorticoid regulated kinase 1) (99). STRING interaction network analysis of these genes highlighted migration and cell cycle regulation as key determinants of the capillary phenotype. Identification of *PRX* as a hallmark of the capillary endothelial cell phenotype is interesting, since it is primarily recognized for its contribution to myelination in nerves. This is not the only gene previously thought to be restricted in its expression to neurons that is found in capillary endothelial cells, as highlighted by evidence that lung endothelium expresses tau and produces Aβ (see sect. 3). Evidence that *PRX* is expressed in capillary endothelium as a determinant of the phenotypic specialization was independently provided by immunofluorescence and immunostaining from Schupp et al. (99) and *The Human Protein Atlas* (https://www.proteinatlas.org/ENSG00000105227-PRX/tissue/lung), respectively, wherein PRX protein abundance is shown to be high. The function of lung capillary PRX is presently unknown. However, brain capillary endothelial cells also express *PRX*, where its PDZ domains have been incriminated in barrier integrity (103). As indicated above, lung capillary endothelium exhibits a highly restrictive barrier; perhaps PRX contributes to this enhanced barrier property.

Venous endothelial cell specification was identified by expression of four genes, including *NR2F2* (Nuclear receptor subfamily 2 group F member 2; alternate name: COUP Transcription Factor II), *VCAM1* (vascular cell adhesion molecule 1), *ACKR1* (Atypical chemokine receptor 1), and *SELP* (P-selectin) (99). A notable commonality among these genes is their link to inflammation. Postcapillary venules sense the proinflammatory environment in the airway and the circulation, they are an important physiological site of permeability, and they contribute to circulating cell transmigration (104–109). Thus, the gene profile matches the recognized physiological function of postcapillary venules (FIGURE 3).

A notable finding from the Schupp et al. analysis is that human and mouse endothelial cell populations have broadly conserved features in regard to their phenotype specification (99). Similar scRNAseq results in human and mouse discriminated among pulmonary arterial, capillary, and venous phenotypes. One example of this point is that capillary endothelial cells do not possess Weibel–Palade bodies, as highlighted previously (48, 81, 82). Similarly, certain populations of human and mouse capillary endothelial cells (see sect. 2.4) do not express mRNA for *VWF* (von Willebrand factor), *SELP* (P-selectin), and *END1* (endothelin-1). Whereas a global fingerprint for endothelium was conserved across species, rodents and humans also displayed distinctions in gene expression. For example, *Car4* expression is an excellent marker for some but not all populations of capillary endothelial cells in mice (in aerocytes but not in general capillary cells; see sect. 2.3), yet it is expressed in all capillary endothelial cell populations in humans (99). *Vwf* expression is absent in lung capillary endothelia in mice, yet it is expressed in some capillary endothelial cells (i.e., general capillary) in humans. Thus, although species-specific differences in gene expression are detectable, in context, humans and mice appear to utilize a similar genetic program to specify endothelial cell phenotype.

### 2.3. Lung Endothelial Cell Microheterogeneity and MAPT

In the past two decades, considerable evidence has revealed the marked heterogeneity among endothelial cells that align the pulmonary arteries, capillaries, and veins. However, even within a vascular segment, endothelial cells exhibit variable functions that have physiological consequences. Such “microheterogeneity” has been previously recognized, based upon anatomical and physiological analyses.

In 1961, Majno and Palade reported the effect of histamine and serotonin on vascular permeability. Using light and electron microscopy, they provided evidence that these inflammatory agonists induce transient intercellular gaps in the systemic circulation as a pathway for fluid and protein permeability (110, 111). The leak site was shown to be across veins, mostly in venules ranging in size from 20 to 30 µm in diameter. Capillaries, as they described them, did not exhibit a histamine- and serotonin-induced increased permeability, consistent with the microheterogeneity described above. Recent scRNAseq data support this physiological observation; histamine receptors are not detected in capillary endothelia but are found in venous endothelium (99). Even within the venous system, however, not every cell-to-cell junction was disrupted, suggesting there is heterogeneity in the response to inflammatory stimuli. This cell-to-cell variability has been a consistent finding, as highlighted by the pulmonary endothelial cell permeability response to agonists targeting the extra-alveolar and alveolar segments, respectively (3, 46, 67).

Similar microheterogeneity has been revealed with regard to endothelial proliferation. This came to light when Schwartz and Benditt (112) resolved endothelial cell clonal niches within the wall of the aorta. They found that replication-competent cell clusters were responsible for endothelial cell proliferation, whereas a majority of cells within the vessel did not replicate. Supportive work was presented in the pulmonary circulation. Ki-67-positive cell clusters were seen in localized vascular sites of vessels undergoing pathogenic remodeling, as in the arterioles that are developing occlusive neointimal lesions (113). The Yoder laboratory examined endothelial cell replication competence as a functional determinant of endothelial progenitor cells. Their work revealed that circulating endothelial cells and conduit endothelial cells, including cells obtained from the umbilical vein, aorta, and pulmonary artery, all possess a minor number of replication-competent cells that rapidly grow from single cells (114, 115). Approximately 10% or less of these cells can grow from a single cell to large colonies in 2–4 wk. Reseeding the rapidly expanding colonies into single cells recapitulates the growth hierarchy, where, again, only a minor fraction of cells give rise to large colonies. These data have been interpreted to mean that a minor fraction of the endothelial cell population exhibits progenitor-like characteristics, possessing an intrinsic ability to regenerate different endothelial cell populations. The minor fraction of replication-competent cells represents a form of microheterogeneity within the population.

Remarkably, cells isolated from the lung microcirculation that express typical endothelial cell markers, like CD31, and are *Griffonia* positive have a high percentage of replication-competent cells within the single-cell cloning assay, especially compared with cells obtained from conduit vessel segments (75, 116). Even after single-cell cloning, these cells retain their differentiated microvascular phenotype. They continue to interact with *Griffonia* lectin and form a restrictive barrier property. They produce blood vessels in Matrigel in vivo, and the blood vessels that are formed tend to be capillary-like blood vessels. These cells are capable of reseeding the capillary segments of decellularized lung scaffolds, indicating that they are capable of homing to their site of origin (117, 118). Lung capillary endothelial cells seem to be a vascular niche that is enriched with replication-competent progenitor cells.

A molecular basis for microheterogeneity within the lung capillary niche was resolved in the scRNAseq reported in the lung endothelial cell atlas (93, 99). Two distinguishable capillary endothelial cell populations were found based on scRNAseq data, including one that is referred to as general capillary and another referred to as an aerocyte phenotype (93). General capillary endothelial cells are the least specialized endothelial cell type as evaluated by the number of genes specifically expressed. Therefore, the genes conserved between human and mice are limited in this cell population. General capillary endothelial cells are characterized by their expression of *GPIHBP1* (Glycosylphosphatidylinositol Anchored High Density Lipoprotein Binding Protein 1), *MCF2L* (MCF.2 Cell Line Derived Transforming Sequence Like), *ITGA6* (Integrin Subunit Alpha 6), *PTPRK* (Protein Tyrosine Phosphatase Receptor Type K), *ADGRE5* (Adhesion G Protein-Coupled Receptor E5), *ZNF608* (Zinc Finger Protein 608), *EPAS1* (Endothelial PAS Domain Protein 1), and *NFIB* (Nuclear Factor I B). General capillary endothelial cells are thought to play central roles in blood vessel development and vascular maintenance, including regulation of barrier integrity. Notably, they contribute to innate immunity and immune surveillance, illustrating the importance of this vascular niche in sensing and responding to the inflammatory status of the parenchyma (FIGURE 3).

In contrast, the aerocyte capillary population was identified based upon the expression of *EDNRB* (Endothelin receptor type B), *TBX2* and *TBX3* (T-box transcription factor 2 and 3), *CDKN2B* (Cyclin dependent kinase inhibitor 2B), *APLN* (Apelin), *RCSD1* (RCSD Domain Containing 1), and VEGF receptor *KDR*. These genes illustrate a role for aerocytes in gas exchange, and they also highlight the relevance of this cell type to development of the lung alveolar-capillary barrier. They would be consistent with a differentiated, growth-inhibitory state of the gas exchange barrier yet important to leukocyte trafficking. The aerocyte would appear to fulfill an essential role in formation of the blood-gas barrier (FIGURE 3).

To assess the anatomical orientation of the general capillary and aerocyte endothelium, respectively, within the lung parenchyma, Gillich and colleagues (93) generated *Aplnr-CreER;Rosa2Confetti* and *Apln-CreER;Rosa2Confetti* reporter mice. Analysis of these mice revealed a nonoverlapping orientation of the *Aplnr*- and *Apln*-expressing cells within the alveolar-capillary plexus. Whereas general capillary endothelial cells were associated with underlying fibroblasts and pericytes in the thick region, aerocyte endothelial cells were adjacent to type I epithelial cells in the thin region of the blood-gas barrier. General capillary endothelial cells were smaller in size and greater in number than the aerocyte population, yet the surface area of the aerocyte was much greater. In total, each of these cell types contributes equally to the lung capillary surface area. Thus, two separate cell types were identified, each with a distinct anatomical locale within the lung microcirculation.

Genetic evidence suggested that aerocyte endothelial cells are quiescent in nature, with a low replication rate (93). To test this idea empirically, the constitutive turnover rate was analyzed by cumulative labeling of 5-ethynyl-2′-deoxyuridine (EdU) over a 6-wk period. The aerocyte population displayed almost no apparent replication. In contrast, EdU incorporation was seen in a small number of general capillary endothelial cells. To examine whether each cell type responds to lung injury with an increase in replication rate, elastase was introduced into the airways, leading to destruction of the alveolar-capillary membrane. In response to this injury, a substantial increase in EdU^+^ cells was seen in the general capillary cells 1 and 6 wk after injury, yet no EdU incorporation was observed in the aerocyte endothelia. These data were interpreted to mean that the general capillary cells represent the progenitor cells within the lung capillary network. Given the high number of general capillary endothelial cells compared with aerocytes (93), this observation is consistent with the interpretation that lung capillary endothelial cells are enriched with progenitor cells (75).

Studies were performed to examine when in lung development these cell populations arise. Both cells were present in the pseudoglandular phase of lung development, a time period in which exuberant expansion of the capillary network and alignment of capillaries with the arterial and venous networks occurs (93). Both cell types differentiated from a bipotent progenitor cell. The general capillary cell was capable of self-renewal of the general capillary cell and also capable of giving rise to differentiated aerocyte endothelia. This concept is consistent with prior studies highlighting the prominent single-cell replication competence of *Griffonia*-positive capillary endothelial cells (75).

**FIGURE 4.**
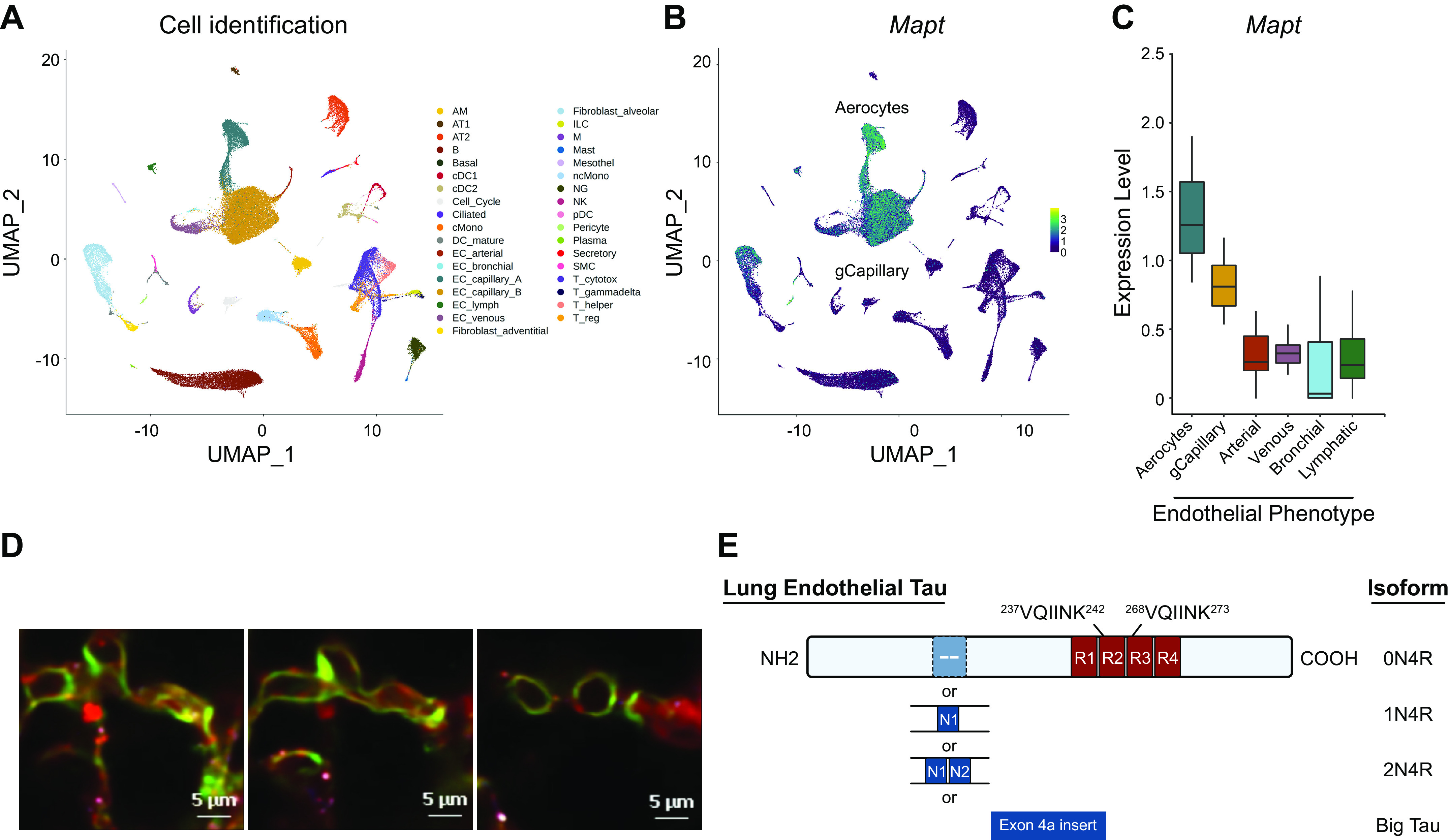
Microtubule-associated protein tau (*Mapt*/*MAPT*) is expressed in lung capillary endothelium of rodents and humans. *A*: single-cell RNA sequencing (scRNAseq) analysis of rodent and human lung reveals cell populations that can be analyzed for discrete archetypes. Analysis is representative for data from 6 repositories at 5 different sites (99). UMAP, uniform manifold approximation and projection. *B*: UMAP analysis highlights expression of tau in lung endothelial cells, including expression in general capillary endothelial cells and aerocytes in the mouse. Quantitation of tau-expressing cells shows expression in variable endothelial cell populations, including capillary endothelia. *C*: quantitation of *Mapt* scRNAseq in lung endothelial cell phenotypes is shown, illustrating highest expression in capillary endothelia, i.e., aerocytes and general capillary (gCapillary) cells. The *y*-axis on the quantitation graph refers to values that are normalized to 10,000 transcripts per cell and are natural log transformed. The expression levels were averaged per subject and per cell type. *D*: capillary endothelia express tau in the mouse, as demonstrated with the tau reporter mouse [B6.129S4(Cg)-*Mapt*^tm1(EGFP/Klt)^/J]. Serial sections of thick sections of a mouse lung were imaged by multiphoton microscopy. Capillaries in the alveolar-capillary membrane are shown. Green fluorescence indicates tau-expressing cells. The fluorescence illustrates that tau expression is present within capillaries but not in adjacent type I epithelial cells. See also Refs. 26, 31. *E*: cloning of the lung and endothelial tau reveals expression of at least 4 isoforms that are highly homologous with brain isoforms, including 0N4R, 1N4R, 2N4R, and big tau isoforms (31). Figure created with BioRender.com, with permission.

Few genes were expressed by all of the lung capillary endothelial cells examined. One of these ubiquitously expressed genes, *Mapt* (microtubule-associated protein tau), is best known for its role in stabilizing microtubules in neurons (93). However, emerging evidence indicates that it is also expressed in organs peripheral to the brain (119), including the lung (26, 31). scRNAseq analysis revealed that *Mapt* is most prominently expressed in the endothelium, alveolar fibroblasts, and pericytes (99), especially in the mouse lung. scRNAseq analysis is inconclusive in human samples and requires additional study (99). This work has been substantiated by different expression atlases, and it is supported by regional analysis of tau expression using a tau reporter mouse [B6.129S4(Cg)-*Mapt*^tm1(EGFP/Klt)^/J] (FIGURE 4). In the latter example, the tau gene has been excised and enhanced green fluorescent protein (EGFP) has been introduced into exon 1, resulting in green fluorescence of any tau-expressing cell. As seen in FIGURE 4, capillary endothelial cells ubiquitously possess green fluorescence. Moreover, CD31- and *Griffonia*-positive capillary endothelial cells studied in vitro express tau, as detected by RT-PCR cloning, proteomic analysis, and Western blotting (26–28, 30, 32–34, 120, 121). Analysis of microtubule architecture illustrates a role for tau in stabilizing microtubules, demonstrating an important regulatory property of endothelial cell tau (30, 120–122). Full-length cloning of rodent lung and endothelial cell tau has demonstrated that lung capillary endothelial cells express at least four tau isoforms, including the 0N4R, 1N4R, 2N4R, and big tau variants (31). Thus, lung capillary endothelia are comprised of at least two distinct cell populations, yet *Mapt* represents a gene central to the function of each.

### 2.4. Lung Capillary Endothelium: Amyloids and Innate Immunity

**FIGURE 5.**
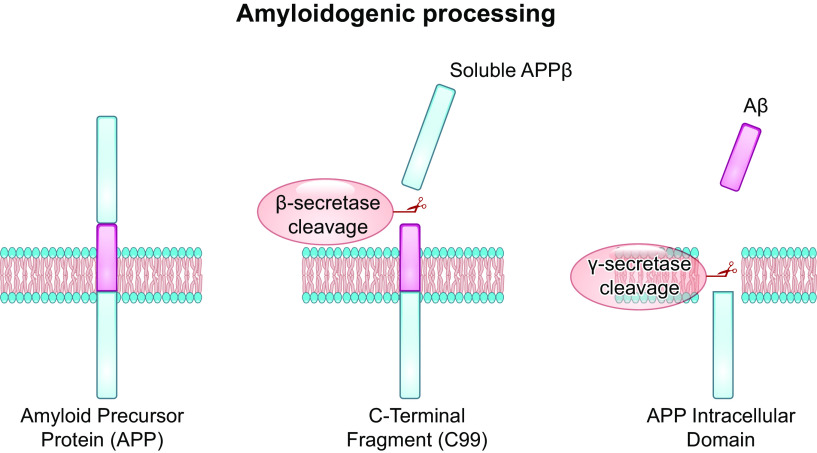
Schematic representation of amyloid precursor protein (APP) processing, leading to the generation of Aβ (adapted from Ref. 139, with permission from *Acta Pharmacologica Sinica*). Amyloid precursor protein is a transmembrane protein. β-Secretase cleaves APP in an endosomal fraction, resulting in the production of soluble APPβ and COOH-terminal (C99) fragments. Endosomal recycling to the plasma membrane enables secondary cleavage of the C99 fragment by the membrane-associated γ-secretase complex, producing Aβ and the APP intracellular domain. Aβ is released outside of the cell. The Aβ that is produced can be variable in size, depending upon the site where γ-secretase cleaves. The most common Aβ monomers produced are Aβ_1-40_ and Aβ_1-42_. Figure created with BioRender.com, with permission.

**FIGURE 6.**
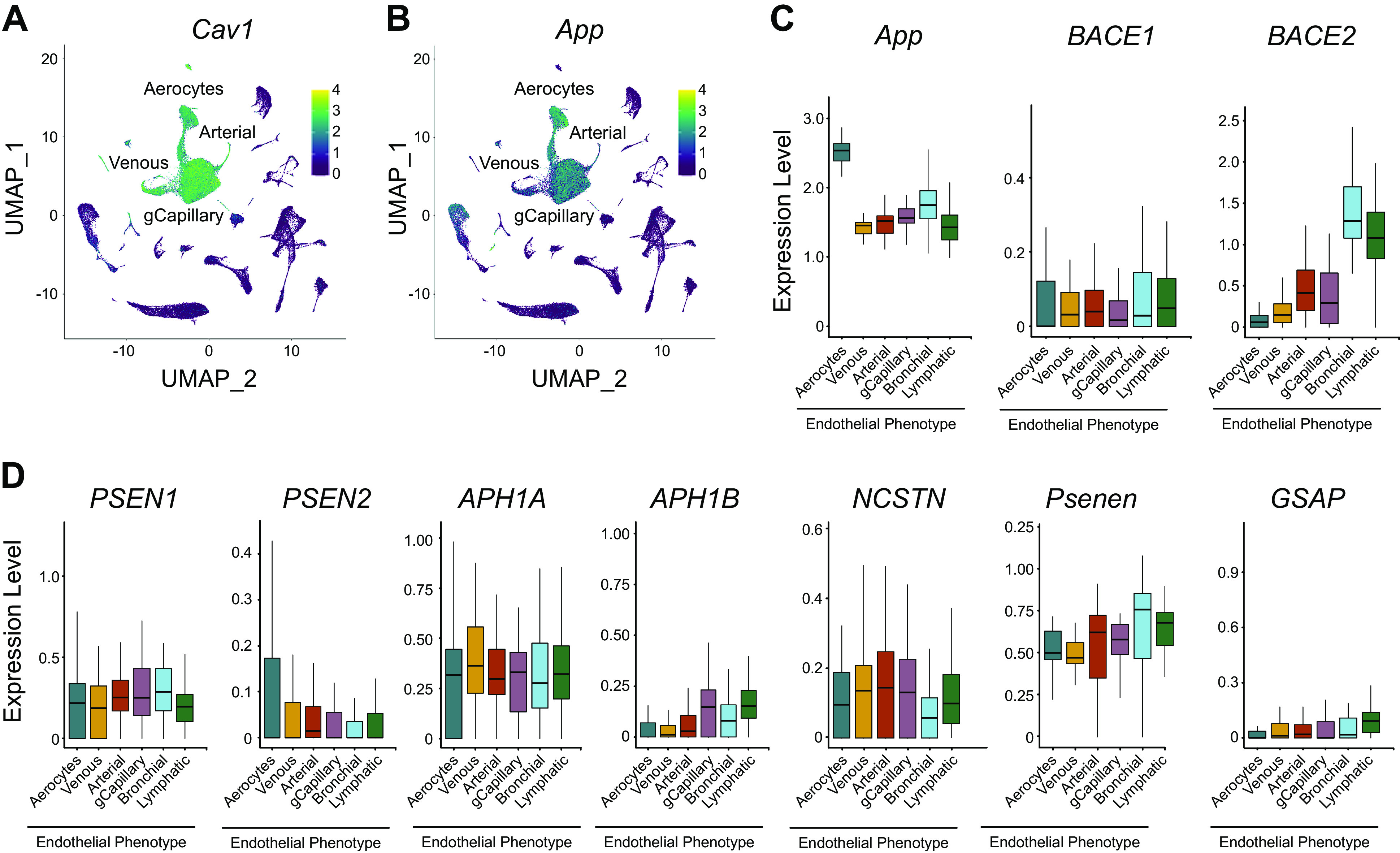
Lung endothelium expresses amyloid precursor protein and the β- and γ-secretases (99). *A*: uniform manifold approximation and projection (UMAP) analysis reveals high caveolin-1 (Cav1) expression in endothelial cell phenotypes, including arterial, venous, and capillary endothelia (99). Both aerocyte and general capillary (gCapillary) endothelia express caveolin-1. Mouse data are shown. *B*: UMAP analysis of amyloid precursor protein (App) reveals substantial expression in lung endothelial cells in both mouse and human (not shown). *C*: both β-secretase 1 (BACE1) and 2 (BACE2) are found in endothelial cells. β-Secretase 2 is most prominently found in bronchial and lymphatic endothelia. Human data are shown. *D*: human γ-secretase subunits are seen in lung endothelia. Presenilin enhancer was not found in the human single-cell RNA sequencing (scRNAseq) (PSENEN) yet was seen in mouse (Psenen); PSENEN was detected in a very low number of endothelia by RNAseq in the human protein atlas (https://www.proteinatlas.org/ENSG00000205155-PSENEN/tissue/lung). *y*-Axis on the quantitation graph refers to values that are normalized to 10,000 transcripts per cell and are natural log transformed. The expression levels were averaged per subject and per cell type. Highlighted genes include Caveolin 1 (*Cav1*), Amyloid Precursor Protein (*App*), Beta-Secretase 1 (*BACE1*), Beta-Secretase 2 (*BACE2*), Presenilin 1 (*PSEN1*), Presenilin 2 (*PSEN2*), Aph-1 Homolog A, Gamma Secretase Subunit (*APH1A*), Aph-1 Homolog B, Gamma-Secretase Subunit (*APH1B*), Nicastrin (*NCSTN*), Presenilin Enhancer, Gamma-Secretase Subunit (*Psenen*), and Gamma Secretase Activating Protein (*GSAP*).

A notable finding from the lung endothelial cell atlas is that capillary endothelial cells express several genes important for innate immunity (99). A significant role for capillary endothelium in the innate immune response to lower respiratory tract infection and the acute respiratory distress syndrome is recognized (123–129). Studies of the lung microbiome have revealed that the distal airway may not be the sterile environment that it was once thought to be (130–132). In addition, blood microbiome studies suggest that the blood environment may not be the sterile environment that it was once thought to be (133–138). Since the lung capillaries are in contact with blood from all of the cardiac output, and since they are intimately associated with alveolar epithelium, the bidirectional communication between epithelial and endothelial cells conveys information regarding the inflammatory status of each compartment, illustrating the importance of capillary endothelium in immune surveillance. As part of its innate immune function, capillary endothelium may contribute to the antimicrobial properties of the distal airway and the circulation. The normal physiological function of Aβ has been incompletely understood. However, there is an increasing appreciation that it is an antimicrobial peptide. Production of Aβ requires the coordinated activity of two multiprotein complexes, the β- and γ-secretases (FIGURE 5). Capillary endothelia express all of the proteins that make up these secretases (FIGURE 6). β- and γ-secretases sequentially cleave amyloid precursor protein to generate Aβ variants. Amyloid precursor protein is a single-pass transmembrane protein that is implicated as a modulator of synaptic formation (140), plasticity, memory (141), and iron transport (142). Amyloid precursor protein is expressed in brain as multiple alternatively spliced isoforms ranging from 639 to 770 amino acids (143, 144) and in endothelial cells as two isoforms of 751 and 770 amino acids (145). The production of Aβ from amyloid precursor protein is a multistep process that occurs because of the activity of the abovementioned family of enzymes called secretases, and the cleavage can result in the production of either nonamyloidogenic or amyloidogenic forms of Aβ (139). In the nonamyloidogenic pathway, amyloid precursor protein can be directed from the trans-Golgi network to the plasma membrane at the surface of the cell or within the endosomal pathway (144). Amyloid precursor protein is first cleaved by α-secretase, resulting in the release of a soluble portion from the cell surface and the retention of a COOH-terminal membrane-associated fragment. The cleavage site for α-secretase is within the region of amyloid precursor protein that is important for formation of amylogenic forms of Aβ, and, as such, the final end products generated by this mechanism exhibit nonamyloidogenic properties (139). The COOH-terminal fragment then is cleaved by γ-secretase to produce a 3-kDa soluble peptide and a residual intracellular domain (146, 147). Aβ produced by the nonamyloidogenic pathway may have neuroprotective effects (148).

In contrast, amyloidogenic processing of amyloid precursor protein involves an initial cleavage by β-secretase (rather than α-secretase) followed by γ-secretase action to produce secreted peptides ranging in size from 30 to 51 amino acids, with Aβ_40_ being the most abundant and Aβ_42_ exhibiting the most deleterious effects (149, 150) (FIGURE 5). The intracellular domain that results from amyloid precursor protein processing translocates to the nucleus, where it is thought to regulate gene expression by modulating the production of important microRNAs (151). The mechanisms that result in the conversion of the production of nonamyloidogenic Aβ to amyloidogenic Aβ are not completely defined.

The specific enzymes involved in the processing of amyloid precursor protein into Aβ_40_ and Aβ_42_ include the β-secretase, called β-site amyloid precursor protein cleaving enzyme 1 and 2 (BACE1 and BACE2; FIGURE 5 AND FIGURE 6), and the γ-secretase complex. Amyloid precursor protein is initially cleaved extracellularly by BACE1 to yield the secreted portion of the amyloid precursor protein molecule plus a 99-amino acid COOH-terminal transmembrane fragment (C99) (152). C99 is subsequently acted upon by the γ-secretase complex, which cuts imprecisely within C99 to form 30- to 51-amino acid-long fragments, including Aβ_40_ and Aβ_42_ (149). The γ-secretase complex is transmembranous and is composed of four individual subunits called presenilin, nicastrin, presenilin enhancer 2, and anterior pharynx-defective1 (APH1). Presenilin is the catalytic component of the complex, and the other subunits are involved in substrate recognition and may have regulatory functions (153). A cytosolic protein called the gamma secretase activating protein assists with amyloid precursor protein processing (154). Gamma secretase activating protein achieves this by binding to both the presenilin subunit of the γ-secretase complex and the C99 fragment of amyloid precursor protein, stabilizing the complex and allowing final production of Aβ (155, 156).

The lung endothelial cell atlas reveals discrete Aβ processing pathways among capillary endothelial cells and the adjacent type I and type II epithelial cells (FIGURE 6) (99). Amyloid precursor protein expression is prominent in all endothelial cell populations, the alveolar epithelium, alveolar macrophages, and monocytes. *BACE1* is expressed in the capillary endothelial cells and alveolar epithelium, although it is at lower levels than what is seen with amyloid precursor protein. It is significant that although the β-secretase enzymes may not cleave amyloid precursor protein in mouse, because of three amino acid substitutions in the target domain, other enzymes, like caspase-1, δ-secretase, and meprin, may substitute for this β-secretase activity (157–163). Subunits of the γ-secretase complex, including PSEN1, NCSTN, APH1A, and PSENEN, are expressed in endothelial cells, yet they are expressed at lower levels and less specifically than amyloid precursor protein. The γ-secretase complex subunits are also found in alveolar epithelial cells, alveolar macrophages, and monocytes. APH1B is either not expressed or expressed at low levels in each of these cell populations, suggesting that APH1A is the predominant homolog utilized by the γ-secretase complex in the lung parenchyma. Amyloid precursor protein and the secretase complexes associate with caveolin-1 within endothelial cell caveolae, and endothelial nitric oxide synthase induces amyloid precursor protein expression (164). In contrast, gamma secretase activating protein is prominently expressed in the alveolar epithelial cells, alveolar macrophages, and monocytes and may be found at low levels in the capillaries. Thus, lung endothelium expresses amyloid precursor protein and has the necessary enzymatic arsenal to generate Aβ. What is known regarding production of both antimicrobial and cytotoxic variants of Aβ and the role infection plays in driving Aβ production are discussed in sects. 3.5 and 4.2, respectively.

## 3. AMYLOIDS

**FIGURE 7.**
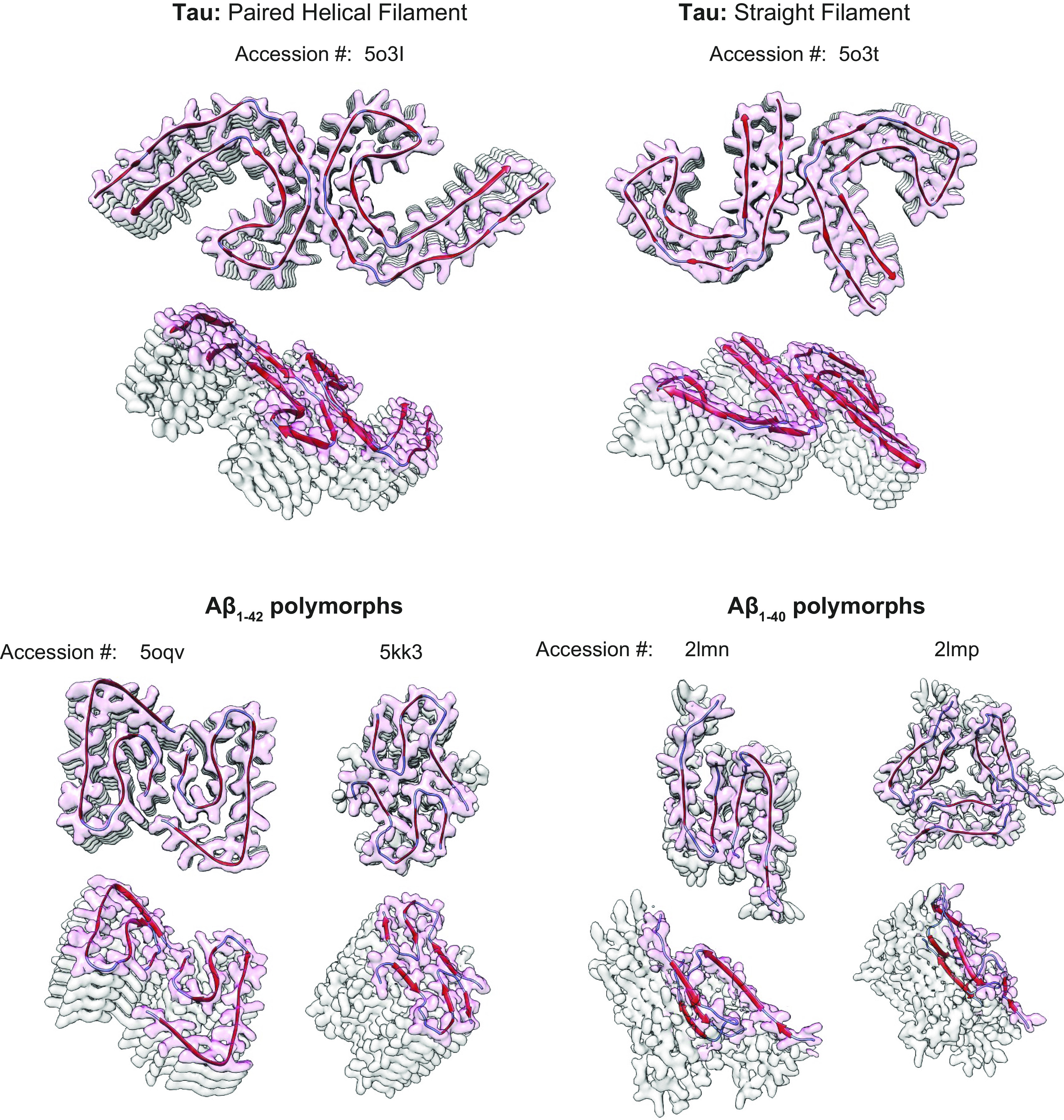
Examples of tau and Aβ fibrils, shown as space-filling models filtered to 4-Å resolution (adapted from Ref. 179, with permission from *Nature Reviews Molecular Cell Biology*). The individual protein/peptide subunits are shown in red to illustrate how protofilaments pack into fibrils. *Top*: tau can assemble into paired helical (*left*, Ref. 169) and straight (*right*, Ref. 169) filaments and stack into ordered fibrils. *Bottom*: both Aβ_1-42_ and Aβ_1-40_ can form amyloid structures. The *left* (180) and *right* (181) structures of Aβ_1-42_ were formed with distinct growth conditions, highlighting that environmental conditions can influence structure. The *left* and *right* structures of Aβ_1-40_ were obtained with the same solutions, although the Aβ_1-40_ peptide in each case originated from distinct propagated seeds (182). This collection of tau and Aβ structures illustrates the idea that variable conformations, or polymorphs, can arise during the formation of amyloids, and these various structures may have distinct activity and/or function. The original data can be accessed via the designated accession numbers.

As highlighted in sect. 2.4, lung endothelium expresses both tau and nonamyloidogenic and amyloidogenic pathways for amyloid precursor protein processing. Endothelial tau plays an important role in microtubule stability relevant to barrier integrity (33, 120, 121). However, lower airway infection can also target endothelial cell tau, converting the physiologically relevant monomeric form into an oligomer(s) that is cytotoxic to the host (26–29, 31, 32, 34). Tau oligomers can acquire an amyloid structure, characterized by the presence of β-sheets between tau stacks, although the amyloid terminology more commonly refers to Aβ (165–178) (FIGURE 7). Whereas the physiological function of tau is described, the physiological function of both nonamyloidogenic and amyloidogenic amyloid precursor protein processing is still incompletely understood. A significant body of evidence supports a role for Aβ in antimicrobial surveillance (reviewed in Refs. 183, 184). Both tau and Aβ “amyloids” can be generated by lung capillary endothelium, as is seen after lower airway infection.

### 3.1. Definition: History of “Amyloid” Discovery

Amyloids were first reported in a collection of >3,000 autopsy reports compiled by Theophili Beneti in 1699, the *Sepulchretum sive Anatomia Practica*. These reports spanned from Hippocrates to Beneti’s time and, beginning in 1639 with Nicolaus Fontanus’ report, included descriptions of spleens filled with white stones or organs so hard they were almost impossible to cut (185). Upon cutting, hard spleens were said to have produced a sound “…like the cutting of spongy timbers.” This phenomenon was also documented in livers, vessels, kidneys, and brains (185–189). Amyloid accumulation within organs was most commonly described in these reports as “waxy” or “lardaceous” (190). In 1838, Matthias Schleiden, who is responsible for the “cell theory,” stained this substance with iodine and sulfuric acid. Schleiden observed results similar to those obtained via the staining of the starch cellulose with the same stain (187) and subsequently called the substance “amyloid,” derived from *amylum*, which is the Latin word for starch (186, 191).

It was not until 1854 that Rudolf Virchow’s investigation of waxy inclusions within the central nervous system brought amyloid to the attention of physician-scientists (188, 192). Virchow, who is recognized as the “father of pathology” (190), had initially proposed that “corpora amylacea,” or inclusion bodies he frequently found in the brain and spinal cord of autopsied patients, were primarily comprised of cellulose or starch (188, 192–194). Using the iodine-sulfuric acid staining method optimized in 1847 by Harting to differentiate cellulose from other starches, Virchow discovered that stained cerebral, midbrain, and spinal cord slices exhibited a strongly positive reaction more akin to cellulose than starch. The term “waxy” continued to be preferred to describe the pathological morphology of amyloids by the Edinburgh School of Pathological Anatomy, and “lardaceous” was the classification proposed by the French School. Virchow rejected both classical designations and adopted Schleiden’s term “amyloid,” to great debate (187, 189, 192–194). He proposed that amyloid was more similar to cellulose in its morphology and staining characteristics, with negligible similarity to lipids, and therefore was neither waxy nor lardaceous (187, 188, 192).

Later that year, Virchow (194) distinguished amyloid corpora amylacea from nonamyloid bodies, which he termed *Hirnsand* (“brain-sand”), and noted that “true” corpora amylacea were primarily found in the ependymal region of the neural ventricles. These findings were corroborated by the pathologist Carl von Rokitansky of Vienna in a personal letter to Virchow (193). Virchow defended his choice of the term “amyloid,” as he noted that although starch quickly stained dark blue with iodine alone, amyloid would not. In fact, the amyloid corpora amylacea would only achieve a “beautiful violet” color after the addition of concentrated sulfuric acid (187, 194). Today, corpora amylacea are understood to be glycoproteinaceous lamellar inclusion bodies (195, 196) that often contain amyloid and have been described in both the lung (197) and prostate (198), as well as the brain. Within the brain they are known to increase with age, but marked increases are most commonly found in the brains of patients suffering from Alzheimer’s (195) or Parkinson’s (196) disease. Despite Virchow’s vehement opposition to the terminology, as well as the discovery in 1859 that amyloid was “albuminoid” and not a lipid whatsoever, the moniker “lardaceous disease” persisted into the early twentieth century (187–189, 191, 192).

The development of silver staining techniques for the microscopic study of neural tissue catalyzed neuroscientific investigation in the late nineteenth and early twentieth centuries, allowing investigators to notice an unusual histopathological phenomenon found during postmortem examination of the brains of elderly dementia and epilepsy patients (199, 200). Although vague descriptions were reported as early as 1888, “round heaps” within the brain that stained strongly with hematoxylin were definitively described in 1892 and called “plaques” by Emil Redlich in 1898. Redlich had noted large numbers of neural plaques in the gray matter of a dementia patient and described them as being of different sizes, with a central nucleus, containing fibrous material, and often located near degenerated ganglion cells (201, 202).

The initial reports of the characteristic plaques of Alzheimer’s disease were made in 1907 by the German physician and assistant professor Oskar Fischer (200). In his landmark study, Fischer examined the brains of 16 patients with senile dementia, 45 neurosyphilis patients, 10 psychotic patients, and 10 control patients. Plaques were found in the cerebral cortex of 12 of the 16 dementia patients yet not within any of the other control brains. Fischer characterized the plaques as “inclusions of unknown origin” consisting of a core and a surrounding corona and associated with many atypical club-shaped neurites that displaced normal tissue, and he provided detailed drawings of these anomalies. He referred to these plaques as *Aktinomyces druse* in reference to the filamentous appearance of an actinomycosis infection. He also described them as *drusige Nekrosen* or appearing to be a “necrotic geode.” Within the field, however, they were most commonly referred to as “Fischer’s plaques” (190, 200, 201).

Shortly after Fischer’s report, Alois Alzheimer described the clinical presentation and postmortem histological findings of the cortex of a presenile dementia patient, Auguste Deter. Alzheimer was the assistant medical director of the Municipal Asylum for the Mentally Ill and Epileptics in Frankfurt, Germany, at the time and had admitted Mrs. Deter and recorded the progression of her case for 2 years before her demise. He noted Fischer’s plaques as “…minute miliary foci which are caused by the deposition of a special substance…[that] can be observed without dye, but it is very refractory to dyeing.” Alzheimer made the novel observation that the neurons seemed to coalesce, ultimately degrading and leaving behind only a “…tangle of fibrils where a neuron was previously located.” This marked the first description of the hallmark neurofibrillary tangles of Alzheimer’s disease. He further noted that the staining pattern of these fibrils was contrary to that of the neurofibrils and concluded that, “The change of the fibrils seems to be a parallel process to the deposition of a pathological metabolic substance in the neuron….” Alzheimer’s work, although technically following Fischer’s description of the neuritic plaque earlier that same year, was unique in that it presented the report of neuritic plaques to be found in conjunction with previously unreported neurofibrillary tangles. Fischer later expanded on his original work in a 1910 paper describing the histological results of 275 brains in which he delineated eight specific stages of neuritic plaque progression (201). Finally, in 1912 Fischer concluded that neuritic plaques were comprised of protein and arose exclusively from neurons in a process of either degeneration or metabolism dysregulation (200, 201, 203).

Although Fischer and Alzheimer made similar observations of neurodegenerative pathology and fundamentally agreed on the origin of Fischer’s plaques, now understood to be primarily composed of Aβ (204–209), they differed in their opinion of the origins of the neurofibrillary tangles. Fischer steadfastly contended that neurofibrillary tangles, now known to consist of pathological tau amyloid, formed stochastically, whereas Alzheimer argued that the tangles resulted from the accumulation of a pathologically dysregulated substance (200–203).

### 3.2. The Amylome

The pathogenic forms of tau and Aβ that were identified by Fischer and Alzheimer are amyloids (FIGURE 7), yet amyloids are now recognized to be widespread in biology. A diverse collection of natively folded or unfolded proteins comprise the so-called “amylome,” which designates proteins capable of forming amyloid fibrils (i.e., amyloidogenic) (210). The amylome includes proteins such as insulin (211, 212), lysozyme (213, 214), atrial natriuretic peptide (215–217), adrenocorticotropic hormone (218), growth hormone (218, 219), and surfactant protein-C (210, 220–222). The collection of proteins that comprise the amylome is vast, although databases of proteins with validated amyloidogenic regions make predicting a protein’s potential to assemble into a functional or pathogenic amyloid an easier task (221). Mammalian amyloidogenic proteins are rich in β-sheets, yet β-sheets alone are insufficient to warrant designation as an amyloid.

Biochemical processes leading to amyloid formation along with detailed structural analysis of amyloids have been recently reviewed elsewhere (179, 223). All amyloidogenic proteins share a core defining characteristic: the proclivity to form a steric zipper motif with another like protein. Nelson and colleagues (224) identified the steric zipper within the yeast protein Sup35 in 2005. Furthermore, there are eight different classifications of steric zippers that are defined by the orientation of polymerizing intermediates (225). Within a steric zipper, polar or aromatic side chains projecting from a double β-sheet tightly interdigitate and form van der Waals interactions or hydrogen bonds with similar protruding side chains from another complementary double β-sheet protein. This interdigitation forms a highly hydrophobic interface, a tight seal, bonding the sheets together. Each component of each sheet is bound to both the segments above and below in the steric zipper interface. In addition, amyloidogenic proteins must have the conformational freedom to polymerize into the cross-β architecture with complementary regions of other amyloidogenic proteins (224–228).

It is the steric zipper that confers the unusual strength and resilience properties that characterize amyloids. The cross-β conformer is stabilized by a notable hydrophobic effect as water is expelled from hydrophobic protein-protein interaction between steric zipper motifs. The hallmark twisting of the amyloid fibril relieves strain during elongation and stabilizes the conformer during polymerization. Amyloids have proven to be highly resistant to denaturation by detergents (212, 229), proteinases (230, 231), and heat (211, 232, 233). However, the extent of stability is influenced by pH and the ionic milieu (234–236). Amyloids are notably resistant to acidic conditions, yet they may be denatured via protracted soaking in undiluted sodium hypochlorite or a strong base (e.g., 2 N NaOH) followed by autoclaving (229, 237).

True amyloids are defined by a distinct quaternary structure, a polymeric architecture designated as a “cross-β spine,” or an amyloid fibril that produces a signature cross-β diffraction pattern (238, 239). The unique cross-β protein structure was described by Geddes et al. (240) in 1968 in his characterization of the silk of the green lacewing fly. The cross-β structure comprises β-sheet-rich amyloidogenic intermediates that polymerize in register, assembling perpendicularly to the axis of elongation (210, 224, 225, 241–244). Out-of-register assembly is possible and has been proposed as a mechanism of cytotoxicity (245). Before polymerization into the hallmark cross-β quaternary conformation, amyloidogenic protein intermediates (e.g., oligomers) are merely amyloidogenic (e.g., they have the potential to give rise to legitimate cross-β amyloid assemblies) and not true amyloids. The biomedical literature commonly refers to “amyloidogenic proteins” as full-fledged “amyloids” for the sake of simplicity, and it has become an accepted convention outside the protein chemistry field.

### 3.3. Amyloidogenesis

Amyloidogenesis is the ordered coalescence of amyloidogenic intermediates into highly ordered, non-covalently bonded protein assemblies that ultimately give rise to the cross-β amyloid fibril (FIGURE 7). This process is often referred to as fibrillization, fibrillogenesis, or the aggregation pathway. In the simplest linear route, β-sheet-rich peptides aggregate into amyloidogenic oligomers (i.e., oligomeric amyloids; the smallest unit to possess the double β-sheet or “amyloid fold”). Oligomers polymerize into protofilaments, then filaments, and finally into the cross-β amyloid fibril. Oligomers with the capacity to initiate polymerization of amyloidogenic proteins into a protofibril, or “nucleate” an amyloid de novo, are frequently referred to as “seeds” (186, 246–251). Amyloidogenic fibrillation generates a sigmoidal curve, and the more seeds and breakage events that occur the greater the rate of fibrillation. Primary nucleation of the amyloid occurs during the lag phase. Fibril elongation occurs during the subsequent growth phase, with the rate dictated by availability of amyloidogenic substrate, agitation, and temperature. The plateau phase is marked by equivalent rates of growth and amyloid turnover and is the saturation phase. Although fibrillation is accelerated via increased temperature and agitation, the capacity of amyloidogenic seeds to retain nucleation capacity is diminished during prolonged heating. Amyloidogenic efficiency is optimized via homotypic polymerization, although heterotypic amyloids are not uncommon (186, 190, 193, 207, 248, 250, 252–255). Cross-seeding occurs when an oligomer, or seed, from one particular type of amyloid (e.g., Aβ) nucleates, or initiates, the fibrillation polymerization while incorporating amyloidogenic intermediates from a different amyloid strain (e.g., α-synuclein) (FIGURE 7). Cross-seeding occurs between amyloid strains as well as between bacterial and mammalian amyloidogenic intermediates or oligomers (203, 243, 250, 256–258). However, amyloidogenesis is rarely a linear process, and off-pathway products frequently result (e.g., amorphous aggregates or nonamyloidogenic multimers/oligomers) that will not permit interaction of complementary steric zipper motifs, thereby making fibrillation impossible (144, 201, 207, 248, 254, 259).

Another nonprotein material that is capable of seeding and cross-seeding growth de novo is crystals. Amyloids have been reported to be chiral colloids that have distinct smectic, cholesteric, and nematic phases dependent on the energy landscape and undergo a fibril-to-crystal conversion. Moreover, amyloids have large aspect ratios rendering them highly anisotropic, conferring them with the property of birefringence when viewed through polarized light. Microcrystalline clusters frequently co-occur with amyloid solutions. Amyloid fibrils have also been reported to grow from the tips of crystals and vice versa. The process of interconversion is still poorly understood but is believed to be thermodynamically mediated (210, 212, 234, 249, 260–263). Within functional amyloids, or amyloids that do not cause disease and perform necessary physiological functions such as the storage and regulation of peptide hormones (e.g., oxytocin) (218, 264), amyloidogenesis is often a reversible process that exists in a dynamic equilibrium in accordance with Le Châtelier’s principle (i.e., dictated by changes in pH, pressure, temperature) (211, 213, 214).

### 3.4. Tau

Tau has classically been considered to be a neuronal protein, although recently it has been identified in cell types peripheral to the brain, including endothelial cells as described above. Tau is a microtubule binding and stabilizing protein (265). Microtubules are hollow cylinders composed of the protein tubulin. Microtubules are dynamic polymers and can readily assemble and disassemble in response to cellular needs by the addition or subtraction of tubulin subunits. Microtubules fulfill multiple roles in cells, including acting as tracks upon which intracellular vesicle transport occurs, serving as spindle fibers responsible for chromosome movements, and, in neurons, serving as the structural element that allows axon stability. In endothelium, microtubules contribute significantly to the restrictive nature of the barrier to water, solutes, and macromolecules (30, 120, 121, 266–273).

The dynamic behavior of microtubules can be regulated by multiple mechanisms, with the major regulatory process being binding by a family of proteins called microtubule-associated proteins (MAPs), of which tau is a member (274–276). Binding of tau to microtubules stabilizes microtubules against disassembly, allowing formation of stable microtubule networks, including the microtubules in nerve axons. The function of tau itself can be modulated by phosphorylation and dephosphorylation, with dephosphorylated tau binding to microtubules and phosphorylated tau dissociating from microtubules, allowing microtubule breakdown (277, 278). Tau hyperphosphorylation in endothelium results in its dissociation from microtubules, causing microtubule collapse that increases permeability (30, 33, 120, 121, 279). Nonetheless, despite its critical role in cells as a modulator of microtubule behavior, knockout animals that lack tau are viable, suggesting that other microtubule-associated proteins have redundant functional capabilities (280, 281).

#### 3.4.1. The molecular basis of tau.

In brain, tau consists of six different isoforms (352–441 amino acids) generated by alternative splicing of RNA transcribed from a single gene located on chromosome 17 (282, 283). Tau is classified as an intrinsically disordered protein and exhibits a modular design that includes zero, one, or two NH_2_-terminal extensions, three or four COOH-terminal microtubule binding domains, and a proline-rich region located between the extensions and the microtubule-binding domains (265, 284). Tau isoforms in the brain represent different arrangements of these modules, with the largest isoform containing both of the NH_2_-terminal extensions and all four microtubule-binding regions whereas the shortest isoform contains neither of the NH_2_-terminal extensions and only three of the microtubule-binding sites, yet all forms are posttranslationally modified to regulate their function. The same is true in lung endothelium; there is near-complete homology between the brain and endothelial cell tau isoforms (31). To date, four tau isoforms have been cloned from rodent lung endothelium, including the 0N4R, 1N4R, 2N4R, and big tau isoforms (31).

#### 3.4.2. Cytotoxic tau variants.

Although a principal physiological function of tau is to stabilize microtubules, it is classified as an intrinsically disordered protein, and its hyperphosphorylation can induce tau to transform into a β-sheet-rich amyloidogenic conformation (FIGURE 7). Two hexapeptides within the microtubule binding repeats (VQIVYK and VQIINK) are capable of forming the steric zipper interface critical for tau amyloidogenesis (285–287) (FIGURE 4). Assumption of the cross-β-sheet conformation assists with assembly into tau fibrils, which eventually form the paired helical filaments characteristic of Alzheimer’s disease (178, 288).

The paired helical filament is a tau lesion observed in neurodegenerative diseases, yet it is the tau oligomer that is thought to be the toxic form of tau. Multiple observations support this conclusion. First, paired helical filaments have been identified in the brains of normal humans, and neurons containing tau tangles can be fully functional in vivo (289). Moreover, the number of neurons lost in the brains of Alzheimer’s disease patients greatly exceeds the number of neurofibrillary tangles measured (290). Another critical observation is that animal models in which tau was overexpressed exhibited considerable neuronal loss accompanied by behavioral abnormalities without formation of neurofibrillary tangles consisting of paired helical filaments (288, 291, 292). Collectively, these observations indicate that assembly of hyperphosphorylated tau into paired helical filaments is not a prerequisite for neuronal cell death. In contrast, several observations support the idea that tau oligomers are the toxic form of hyperphosphorylated tau. As tau becomes hyperphosphorylated, it dissociates from microtubules and binds to additional hyperphosphorylated monomers to form oligomeric aggregates that become detergent insoluble (293). Injection of tau oligomers into brains of mice induced cognitive and behavioral abnormalities (294–296). Moreover, oligomeric tau has been shown to induce the misfolding of endogenous tau and the propagation of misfolded tau between different brain regions, whereas fibrillar tau had no effect (297). This observation supports a prionlike mechanism for toxic tau transmission through the brain (297–300). Finally, injection of antibodies directed against oligomeric tau protected against cognitive decline in animal models of Alzheimer’s disease (294, 301). More recently, clinical trials using antibodies against oligomeric tau as a treatment for Alzheimer’s disease have produced promising results (302).

The pathophysiological mechanisms leading to the transition of physiologically relevant tau monomers into oligomers have not been completely defined. The phosphorylation of tau is a normal cellular process, and, as stated above, it mechanistically regulates tau association with microtubules and microtubule stability. All six brain isoforms of tau can be phosphorylated, and multiple kinases have been identified that phosphorylate tau, including MAP kinase, PKA kinase, and GSK3β, among others (285, 303). Mass spectroscopy has demonstrated that paired helical filaments from the brains of Alzheimer’s disease patients contain up to 20 phosphorylated amino acids, whereas tau in normal brain contains four or five phosphorylated amino acids (304, 305). At present, it is not known how many phosphorylation events, or which amino acids must be phosphorylated, to trigger tau oligomerization (304). Regardless, many of the phosphorylation sites have been mapped to the microtubule-binding repeats, and phosphorylation within these regions decreases the affinity of tau for microtubules (306, 307). As tau dissociates from microtubules, hyperphosphorylation presumably increases the affinity of tau monomers for one another, leading to the formation of tau oligomers. The extent and sites of posttranslational modifications lead to structurally diverse oligomeric variants (165). As stated, these oligomers are thought to be the toxic forms of tau and most likely are also intermediates on the pathway to helical filament assembly. Clearly, much more needs to be learned about physiologically versus pathologically relevant tau phosphorylation events and how these events lead to tau oligomerization.

#### 3.4.3. Cytotoxic tau release and activity.

Once produced, oligomeric tau is released from cells. Tau lacks a signal peptide, so it needs to be released via a specialized secretion event. Tau is found outside the cell, in biological fluids including the bronchoalveolar lavage fluid, blood, and cerebrospinal fluid (26, 28, 29, 308). It can also be resolved in the cell supernatant of cultured neurons and endothelial cells (28, 32, 34, 309). Although the presence of tau in these instances was initially suggested to be due to cell death (310), recent evidence indicates that tau release is a normal physiological process (311) and cytotoxic oligomers can be released before cell death (32, 312). Two separate pools of extracellular tau have been identified, an exosome-associated pool and a larger pool of soluble, free tau (313, 314). The free pool appears to be released in a constitutive manner, which in neurons can be stimulated by neural activity via a mechanism that does not involve the classic rough endoplasmic reticulum (rER) and Golgi pathway (314, 315). Schematically, oligomerization would allow translocation via membrane insertion and disassembly in the extracellular space, with the process being dependent on interactions of tau with phosphatidylinositol 4,5-bisphosphate [PI(4,5)P_2_] and sulfated proteoglycans (316, 317). Secretion of tau via exosomes is proposed to involve capture of cytosolic proteins in internal vesicles that then fuse with multivesicular bodies (318). The multivesicular bodies can then fuse with the plasma membrane and release their contents.

Although multiple mechanisms have been proposed for the uptake of tau oligomers into target cells, the most strongly supported process at present is receptor-mediated endocytosis (288). Studies have shown that the minimal form for uptake and seeding of intracellular aggregation is trimeric tau (319). Recent attempts to identify a receptor for trimeric tau have built upon the observation that the prion protein binds to heparan sulfate proteoglycans during its uptake and propagation (320, 321). Holmes et al. (322) used inhibitors, enzymatic treatment, and genetic knockdown to demonstrate that heparan sulfate proteoglycans are also required for oligomeric tau uptake and propagation in cultured cells and then used an inhibitor to demonstrate that heparan sulfate was also involved in the uptake of injected tau into intact brain. Rauch et al. (323) recently screened for the involvement of known heparan sulfate proteoglycan partners in the tau uptake process and determined that knockdown of low-density lipoprotein receptor-related protein 1 (LRP1) inhibited uptake of tau oligomers into cultured cells and also blocked the spread of tau in an animal model in vivo. These observations identify LRP1 as a common player in the spread of both tau and Aβ lesions (see sect. 5.7.3). In addition to LRP1, evidence indicates that tau oligomers can bind to M1 and M3 muscarinic receptors (324, 325). Binding to these receptors led to prolonged increase in intracellular Ca^2+^, which could ultimately lead to cellular toxicity.

Oligomeric tau may disrupt mitochondrial function, which contributes to cytotoxicity. Microtubules are required for transit of mitochondria to synapses, where the mitochondria generate energy required for synaptic activity. Hyperphosphorylation of tau and assembly into helical filaments can result in physical blockade of axons, disrupting axonal transport leading to cell death (326, 327). However, oligomeric tau also has subtler effects on mitochondrial activity. Stereotaxic injection of tau oligomers directly disrupted complex 1 activity in neurons, impairing ATP production in affected cells and increasing the activity of mitochondrion-associated caspase 9 (296). In addition, it has also been reported that tau disruption leads directly to mitochondrial fragmentation and an increase in membrane permeability (328), providing another possible explanation for the toxic activity of tau oligomerization.

Numerous studies have been performed to investigate the relationship between tau and Aβ, and these studies support a model in which Aβ lies upstream of tau in the pathological events leading to neurodegeneration (329). Specifically, Aβ has been shown to initiate tau hyperphosphorylation and neuronal cell death in cultured neurons (330–332). The proposed mechanism for this is through Aβ-induced activation of kinases responsible for hyperphosphorylation of tau, including GSK3β and MAP kinases (333), JNK kinase (334), and Fyn (329, 335). In vivo, injection of Aβ into the brains of mice expressing a mutant form of human tau resulted in the rapid assembly of paired helical filaments near the injection site (336). The conclusion that tau lies downstream of Aβ and that tau is required for Aβ-induced toxicity was provided by animal models in which tau was either knocked out or depleted. Specifically, when tau knockout mice were crossed with mice overexpressing mutant human amyloid precursor protein, amyloid plaque formation was not decreased in the offspring animals, although memory deficits were prevented (337). In a related study, when double-mutant mice expressing both mutant amyloid precursor protein and presenilin-1 were crossed with tau knockout mice, the offspring were protected against memory loss, neuronal death, synaptic loss, and early death (338). Collectively, these data support a model in which oligomeric Aβ initiates the hyperphosphorylation of tau, leading to neurodegenerative processes.

Although the available literature supports a role for hyperphosphorylation as an essential initiating event that leads to generation of cytotoxic tau variants, it is important to note that an initiating event remains unknown. That is, mechanisms leading to tau hyperphosphorylation are poorly described. In concept, these mechanisms may be generation of ligands that activate kinases driving tau phosphorylation and/or impaired tau-associated phosphatase activity. The mechanisms are a major challenge for future studies.

### 3.5. Beta-Amyloid

Amyloid precursor protein processing results in nonamyloidogenic and amyloidogenic products, as described above (see sect. 2.4). Because of its association with Alzheimer’s disease, the most extensively studied amyloid is Aβ (308). Aβ is the major component of the amyloid plaques that, along with paired helical filaments, are the characteristic lesions observed in brains from patients with Alzheimer’s disease. In addition, Aβ is associated with related neurodegenerative diseases (339) and, more recently, pneumonia (28, 29, 308).

Once formed, Aβ monomers can assemble into different types of aggregates that include oligomers, polymers, and fibrils, with the fibrils having the capacity to assemble further into the amyloid plaques characteristic of brains from patients with Alzheimer’s disease and other neurological disorders (FIGURE 7). The Aβ monomer has not been crystallized, most likely because of an unstructured behavior in solution, and much of what is known about the structure of the monomer has been derived from NMR studies. Nuclear magnetic resonance analyses suggest that the COOH-terminal amino acids of Aβ_42_ provide inflexibility that may contribute to its enhanced capacity for amyloid formation (340). Aβ_40_ and Aβ_42_ are identical to each other, with the exception that Aβ_42_ contains Ile^41^ and Ala^42^, and, as stated above, it has been proposed that these two additional amino acids contribute to the hydrophobicity and double β-sheet-forming characteristics of Aβ_42_, allowing its enhanced fibrillogenic and plaque-forming capacity relative to Aβ_40_ (204, 341, 342). Nuclear magnetic resonance has determined that Aβ is folded into a predominantly α-helical shape, although it does assume a β-sheet conversion in the presence of more hydrophobic solutes that would mimic plasma membrane environments (343).

The structure of the amyloid fibers characteristic of many neurodegenerative diseases is more well defined (FIGURE 7). These fibers, which assemble via a poorly defined process from Aβ monomers, have been the focal point of Alzheimer’s disease research since the amyloid cascade hypothesis was initially proposed (344). According to this model, the deposition of Aβ into amyloid plaques composed of amyloid fibers is the causative event for Alzheimer’s pathology, including the neurotoxicity and dementia characteristic of the disease. The fiber consists of hydrogen-bonded parallel and antiparallel β-sheets (139). These fibers can be readily seen by electron microscopy as long, straight polymer fibers (345) and are characterized by their capacity for intercalating various amyloid-differential dyes, including Congo red and thioflavin T, into the amyloid fold, thereby generating a measurable signature, which allows the identification and quantification of their levels in solution (e.g., via fluorescence) and in tissue sections (e.g., via polarized light-induced yellow-green birefringence) (346). However, the pathogenetic importance of large amyloid polymers and plaques in neurodegenerative diseases has been questioned. Specifically, Aβ accumulation and plaque formation do not correlate with memory loss and dementia (347–349). Moreover, neuronal loss is not observed in several transgenic models designed to mimic human Alzheimer’s disease (349).

A more likely candidate for the toxic species of Aβ is its oligomeric form (350). This conclusion is supported by in vitro, animal, and human studies (351–354). An advantage of small oligomers of Aβ, which may be dimers, trimers, or larger complexes, is that they have the capability of diffusing in tissues, unlike large fibers, which are incapable of diffusion. The capacity to diffuse within tissues would provide a mechanism for spreading of toxic activity. Aβ oligomers, which also exhibit a β-sheet organization (355), can be detected in cell culture supernatants and tissue homogenates with the oligomer-specific polyclonal A11 antibody, although the A11 antibody is not specific for Aβ, and the MOAB-2 antibody among others (356). Regardless of the species of Aβ responsible for neurotoxicity, the causative event leading to production of neurotoxic Aβ is undefined.

Multiple mechanisms have been proposed for explaining Aβ toxicity, and these are discussed in detail elsewhere (139, 345). Briefly, in vitro evidence indicated that Aβ can bind directly to lipid membranes, with membrane association causing intracellular Ca^2+^ fluctuations that may be due either to signal transduction processes or to direct pore formation (357, 358). Multiple studies have also demonstrated that Aβ oligomers can induce oxidative stress in cells (359, 360), with the proposed mechanism being via binding of heavy metals by Aβ, reduction of the metal ions to generate superoxide and peroxide, and then direct action on membrane lipids to induce lipid peroxides and carbonyls (361, 362). In addition to direct pore formation and oxygen radical generation, multiple receptors have been suggested to be involved in the pathological effects of Aβ oligomers, including the glutamine channel receptor (363, 364), the α_7_nAChR acetylcholine receptor (156, 365), and others. Besides leading to cell death, activation of receptors by Aβ oligomers also rapidly disrupts neurotransmission, as demonstrated by abolishment of long-term potentiation (345).

Another potential mechanism of toxicity is direct uptake of Aβ (366). Uptake appears to occur at lipid rafts (367), and intracellular Aβ has been proposed to disrupt mitochondrial function (368–370). Multiple excellent reviews have been published related to Aβ, its assembly, and its mechanisms of action, and readers are directed to those references for further details (139, 345, 350, 359, 371).

Although Aβ has classically been considered a molecule that is cytotoxic to mammalian cells, and the amyloid cascade hypothesis has driven research related to Alzheimer’s disease and other neurodegenerative diseases for several decades, recent evidence suggests that Aβ may have an essential protective physiological role as a component of the innate immune system (reviewed in Refs. 183, 184). According to this model, Aβ is viewed as an antimicrobial peptide that undergoes fibrillization to protect the host brain from invasion by both viral and bacterial infection (372). Fibrillization entraps the pathogenic invader with the fibrils and then permeabilizes cell membranes via direct insertion leading to cell death by disruption of ion homeostasis (358, 373). Evidence to support this role for Aβ as an antimicrobial peptide includes the demonstration that plaques are present in both dementia brain and healthy brain, and in both cases the plaques contain DNA from infectious agents (374, 375), whereas infection by viral and bacterial pathogens induces amyloidogenic Aβ production (183). In addition, overexpression/induction of Aβ in an animal model has been reported to protect against infection by S*almonella typhimurium* (376). In further support of the antimicrobial properties of Aβ, several publications have reported that a common side effect in clinical trials targeting Aβ as a treatment for Alzheimer’s disease is elevated rates of infection in study participants, as would be predicted by specific removal of an antimicrobial peptide (183, 377, 378). Collectively, these data support a model in which a normal physiological role of Aβ is as a component of the innate immune system responsible for combatting infections. Given that Aβ appears to be an essential component of the innate immune response because of its antimicrobial properties, future studies are needed to determine the mechanisms that convert it from a protective antimicrobial peptide to a cytotoxic molecule.

## 4. INFECTIOUS PROTEINOPATHY: CYTOTOXIC LUNG ENDOTHELIAL AMYLOIDS

**FIGURE 8.**
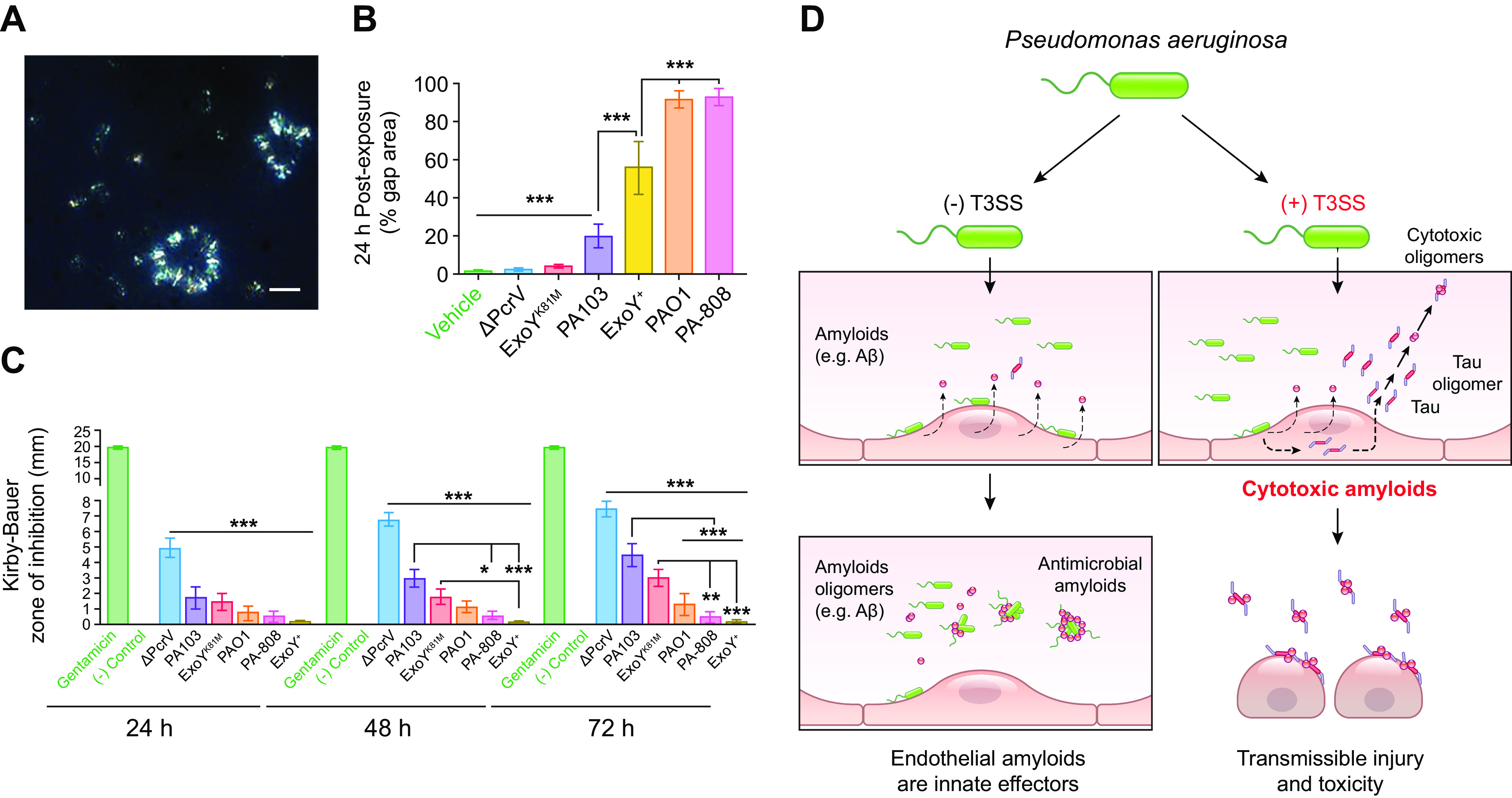
Type III secretion system intoxication converts intrinsically protective endothelial amyloids into prionlike cytotoxins. *A*: Congo red-stained liquid culture of *Pseudomonas aeruginosa* treated with endothelial cell supernatant from T3SS-incompetent (ΔPcrV) infection observed via polarized microscopy to reveal the apple-green birefringence characteristic of amyloids. Scale bar, 50 µm. *B*: confluent microvascular endothelial cells were incubated with vehicle or filter-sterilized supernatants generated from endothelial intoxication with ExoU/T (PA103), ExoY alone (ExoY^+^), or ExoS/T/Y (reference strain PAO1 and clinical isolate PA-808). ExoY-competent strains induce the greatest total area of interendothelial disruption at 24 h after treatment. *C*: whereas infection with T3SS-incompetent ΔPcrV *P. aeruginosa* mutant elicits endothelial amyloids that are not cytotoxic, they are distinctly antibacterial in a time-dependent manner. Infection with ExoY-competent lab and clinical strains, however, markedly diminishes or ablates antimicrobial efficacy. *D*: the schematic demonstrates the type III secretion system-dependent dichotomy of endothelial amyloid function. Lung endothelial releases amyloids including Aβ, both constitutively and in response to infection, that are innate defense peptides. Type III secretion system-competent infection, particularly ExoY intoxication, induces the production and release of cytotoxic tau oligomers, which negate beneficial antimicrobial amyloid activity and instead promote a tauopathy. Adapted from Ref. 34, with permission from *FASEB Journal*. *n* ≥ 7 with 3 technical replicates per independent experiment, repeated-measures or standalone 2-way ANOVA with Bonferroni post hoc; mean ± SE. *P < 0.05, **P < 0.01, ***P < 0.001.

Lung capillary endothelial cells express tau and amyloid precursor protein, as well as the genes relevant to the function of each (see FIGURES 4–6). Whereas a principal function of tau is to stabilize the endothelial cell microtubule network relevant to barrier integrity, the principal role of amyloid precursor protein within the lung’s capillary niche remains incompletely understood. Nevertheless, based upon the known function(s) of Aβ, an important constitutive process of amyloid precursor protein is its contribution to the production of antimicrobial proteins that contribute to immune surveillance of the lower airway and the circulation. Both tau and Aβ are molecular targets of host-pathogen interactions, i.e., contribute to interkingdom communication. Lower respiratory tract infection drives biochemical and functional shifts in the functions of tau and Aβ, resulting in the generation of amyloid variants that are injurious to the host (26–29, 31, 34, 308) (FIGURE 8). As a consequence, cytotoxic variants of tau and Aβ are released from lung endothelium and serve as a source of tissue injury, both locally and peripheral to the lung. Here, we examine what is known about how infectious stimuli drive production of cytotoxic tau and Aβ variants.

### 4.1. Cytotoxic Tau Variants

Lung endothelial cell tau stabilizes microtubules (30, 32, 120–122, 266–268, 270–273). During lower respiratory tract infection, however, monomeric tau is modified by intracellular signaling events that lead to its dissociation from microtubules (30, 33, 272). Tau dissociation promotes microtubule collapse, and microtubule collapse increases permeability. In association with this hyperpermeability, tau is released from endothelium through unknown mechanisms; it is clear that tau release is not simply due to cell lysis (32, 312), yet tau possesses no known secretion sequence, suggesting that it is released through a noncanonical pathway, as described above (see sect. 3.4). Tau that is released from the endothelium during infection is cytotoxic. The structural nature of these infection-elicited cytotoxic tau variants is unknown, yet the phosphorylation status is essential to tau cytotoxicity.

Cytotoxic tau has been recovered from the bronchoalveolar lavage fluid, plasma, and cerebrospinal fluid of pneumonia patients who have ongoing infection due to either bacteria or viruses (26, 28, 29, 308). Moreover, cytotoxic tau has been found in the biological fluids weeks after lung infection, even after effective antibiotic treatment (31); it is notable that the half-life and mechanisms responsible for turnover of cytotoxic tau following infection are unknown. The organisms known to initiate cytotoxic tau production include common causes of nosocomial pneumonia, like *Pseudomonas aeruginosa*, *Klebsiella pneumoniae*, and *Staphylococcus aureus* (26, 28, 308). In addition, organisms like *Morganella morganii* have been shown to drive cytotoxic tau production (26). A comprehensive list of pathogens capable of triggering cytotoxic tau production has not been compiled, yet it appears most likely that virulent organisms are capable of eliciting cytotoxic tau production.

**FIGURE 9.**
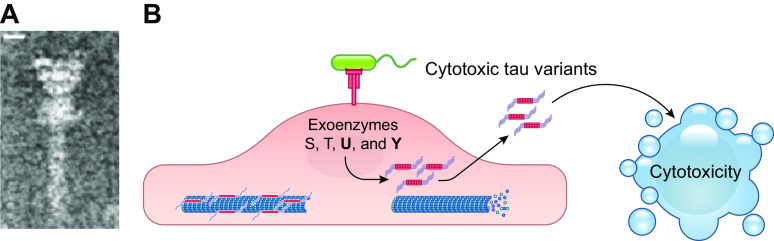
The *Pseudomonas aeruginosa* virulence arsenal and specifically the type III secretion system and its effectors are essential mechanisms of interkingdom communication that drive production of cytotoxic tau variants in endothelium during infection. *A*: ultrastructural analysis of the *Shigella* type III secretion system illustrates the structure of the needle apparatus that translocates exoenzymes from the bacteria to the host cell. The needle extension has a pore that proteins pass through. Once in the host cell the enzymes acquire a tertiary structure, bind to a mammalian cell cofactor, and acquire enzymatic activity. Scale bar, 20 nm. Adapted from Ref. 379, with permission from *EMBO Journal*. *B*: schematic representation of *P. aeruginosa* introducing exoenzymes S, T, U, and Y into the host cell through a type III secretion system. Virulent clinical isolates typically utilize 3 of the 4 exoenzymes in their virulence arsenal, most commonly a combination of exoenzymes S, T, and Y and less frequently a combination of exoenzymes T, U, and Y. Exoenzymes U and Y are bolded because their activity has been mechanistically linked to endothelial tau hyperphosphorylation (26, 28, 31, 32, 34). Once produced, the infection-elicited tau variants are cytotoxic. Figure created with BioRender.com, with permission.

It is notable, however, that the mere presence of bacteria is not sufficient to elicit this response; rather, determinants of bacterial virulence seem to be necessary (FIGURE 8 AND FIGURE 9). This assertion is based upon mechanistic studies using *P. aeruginosa* as a model system. These studies reveal that *P. aeruginosa* infection with bacterial strains lacking the type III secretion system does not elicit cytotoxic tau production and does not cause protracted lung injury (FIGURE 8) (34, 380, 381). In contrast, type III secretion system-competent bacterial strains promote cytotoxic tau generation (FIGURE 9). Four type III secretion system effectors are introduced into host cells, including exoenzymes S, T, U, and Y. Among these, ExoT is the most prevalent exoenzyme (∼100%), followed by ExoY (∼90–95%), ExoS (∼75–80%), and ExoU (∼20%) (382). Most clinical strains isolated from mechanically ventilated pneumonia patients possess three of the four exoenzymes, where the most prevalent combination is ExoS, ExoT, and ExoY (383–386). ExoS and ExoT are both bifunctional enzymes that possess GTPase and carboxy-terminal ADP-ribosyltransferase activities following enzymatic activation by a 14-3-3 protein cofactor (387–389). ExoU has phospholipase activity. Its cofactor is ubiquitin, and it can be activated by phosphatidylinositol 4,5-bisphosphate (390–393). Upon acquisition of enzymatic activity, ExoU causes cell lysis. Clinical strains utilizing ExoS, ExoT, and ExoY elicit release of cytotoxic tau from endothelium (34). ExoU is also capable of initiating production of the transmissible cytotoxin, yet ExoY’s activity appears to most effectively produce the transmissible cytotoxic activity (32, 34). The enzymatic activities of ExoU and ExoY are sufficient to promote cytotoxic tau production, even in the absence of bacteria (32, 33).

Among the type III secretion system effectors, ExoY has emerged as an important bacterial mechanism that drives cytotoxic tau production (34). Once ExoY is introduced into the host cell it acquires a tertiary conformation that is needed for binding its mammalian cofactor. Although the mammalian cofactor was unknown for many years, the Mechold laboratory has recently determined that it is filamentous actin (F-actin) (394–397). Once ExoY binds F-actin it acquires nucleotidylyl cyclase activity that informs cellular responses. The ExoY-dependent cAMP signal leads to tau phosphorylation, microtubule breakdown, cell rounding, and disruption of endothelial cell barrier integrity (33, 272, 279, 381). ExoY belongs to a family of actin-regulated cyclases. Actin-regulated cyclases represent a common virulence mechanism among bacteria; >25 bacteria have been shown to or are predicted to possess actin-regulated cyclases within their virulence arsenal (394). Tau appears to be an important host target of the actin-regulated cyclases.

Whereas ExoY was first found to be an adenylyl cyclase, it later became evident that bacterial soluble cyclases do not just produce cAMP, they produce cGMP and other noncanonical cyclic nucleotide monophosphates (cNMPs), including the purine cNMPs cUMP and cCMP (33, 398–404). ExoY first produces high levels of cGMP in capillary endothelium, followed by high levels of cUMP (33, 402). These studies suggest that in lung capillaries ExoY predominantly generates cGMP and cUMP and secondarily produces cAMP. How ExoY distinguishes which cNMP to produce over what time period has not been determined. Nonetheless, these cNMPs collectively activate protein kinases A and G, and the activation of these enzymes leads to tau phosphorylation as a prelude to generation of cytotoxic tau variants. These studies have led to the idea that tau phosphorylation is a critical step in generation of cytotoxic variants after infection. In support of this idea, global dephosphorylation of tau isolated from lung endothelium after infection abolishes its cytotoxicity to the brain (405).

It is notable that cytotoxic tau is not present under control conditions, i.e., in the absence of infection, including in time control experiments and in cells and animals infected with *P. aeruginosa* mutants possessing catalytically dead ExoY and/or a dysfunctional type III secretion system needle. Similarly, bacterial lysates, bacterial culture medium, uninfected endothelial cell lysates, and supernatant from uninfected endothelial cultures are all without effect. Therefore, *P. aeruginosa* strains/mutants possessing a functional type III secretion system needle and an enzymatically active effector are necessary for the endothelium to produce and release a transmissible cytotoxin. As highlighted above, however, virulent organisms that do not utilize a type III secretion system, like *S. aureus* and *K. pneumoniae*, can elicit cytotoxic tau production; in these instances, the mechanisms utilized by these organisms to elicit production of cytotoxic tau variants are unknown.

Although the tau phosphorylation status is important for its cytotoxicity, the biochemical and molecular nature of this cytotoxic activity is still incompletely understood. It is highly protease resistant (27). For example, 8 h of proteinase K treatment does not inactivate tau cytotoxicity. Cytotoxic activity sediments with 8 h of high-speed centrifugation (150,000 *g*; angular momentum ∼1.14 × 10^12^) (27). It is heat stable, meaning that boiling the cytotoxic tau does not inactivate its cytotoxicity (26, 28, 34). It is inactivated by organic solvents, like diethyl pyrocarbonate, phenol, and hexafluoro-2-isopropanol. These biochemical features are characteristic of prion disease, similar to, but different from, misfolded prion protein (i.e., PrP^SC^). The structural basis of infection-induced cytotoxic tau variants has not been determined.

Prions are transmissible among cells. Transmissibility of the infection-dependent endothelial cytotoxin was tested both in vitro and in vivo (26, 28, 34). The cytotoxic supernatant was prepared and applied to naive cells. After 4 h, before cells exhibited cytotoxicity, the supernatant was removed and replaced. After an additional 16 h, the supernatant was collected, centrifuged, filter-sterilized, and incubated on another population of naive cells. Twenty-four hours later, widespread cytotoxicity was observed. This process was repeated for eight passages, and in each passage the cytotoxic activity was preserved, indicating that the infection-induced endothelial cytotoxin is transmissible and self-replicating. Infection initiates a tauopathy (26).

**FIGURE 10.**
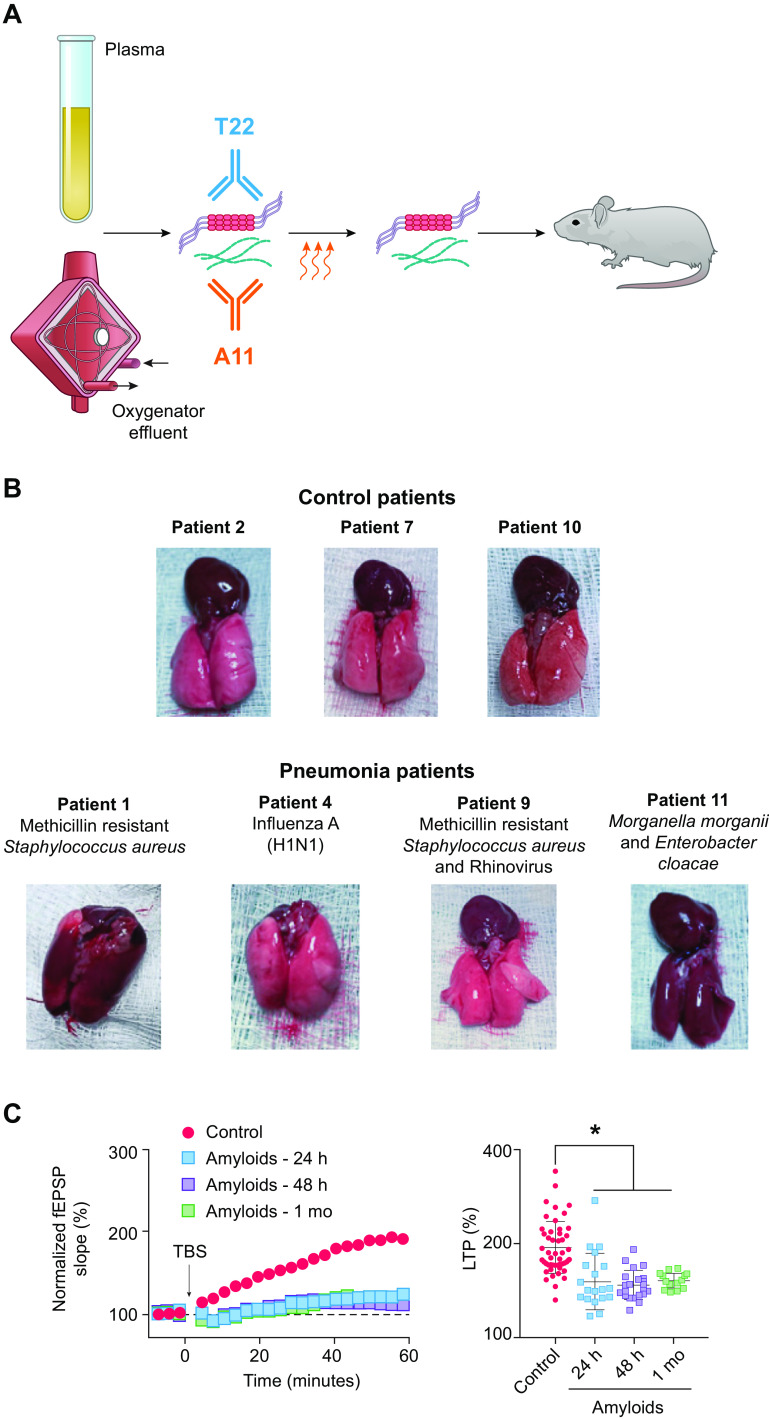
Cytotoxic tau and Aβ variants circulate in the plasma fraction of humans and animals after lung infection and persist even after antibiotic treatment. *A*: plasma fractions isolated from peripheral blood samples or from extracorporeal membrane oxygenator effluents contain cytotoxic variants of tau and Aβ that can be immunoisolated with T22 and A11 antibodies. Introduction of boiled (heat illustration) and purified samples into either the airways or blood of naive animals leads to lung and brain injury (26). *B* and *C*: cytotoxic tau and Aβ variants isolated from the extracorporeal membrane oxygenator effluents of infected, but not uninfected, effluents and introduced into naive rodents (i.e., uninfected) cause lung injury (*B*) and impair hippocampal long-term potentiation (LTP) (*C*). This transmissible end-organ dysfunction is a tau-dependent phenomenon (26, 31). fEPSP, field excitatory postsynaptic potential; TBS, theta burst stimulation. *One-way ANOVA with Tukey’s post hoc test (*P* < 0.001). Figure created with BioRender.com, with permission.

This principle, that cytotoxic tau variants are transmissible and self-replicating, was tested in vivo (26) (FIGURE 10). To test this idea, *P. aeruginosa* was introduced into the airway and blood was collected from the abdominal aorta 48 h after infection. These animals exhibited acute lung injury, a hyperdynamic circulatory state, and impaired hippocampal long-term potentiation, indicating end-organ dysfunction due to the *Pseudomonas* infection. Anti-tau (i.e., T22) and anti-amyloid (i.e., A11) antibodies were used to capture cytotoxic tau and Aβ variants from the plasma fraction. Tau and Aβ were eluted off of the antibodies, resuspended into a small volume of phosphate-buffered saline, and introduced into the airway of a naive, i.e., uninfected, animal. Twenty-four and forty-eight hours later, and again 1 mo after tau and Aβ introduction into the airways, long-term potentiation was tested. Even 1 mo after introduction of tau and Aβ into the airways of a naive animal, lung injury was seen and hippocampal long-term potentiation was suppressed. Thus, cytotoxic tau and Aβ variants are present in the circulation after infection, and when they are isolated from the circulation and subsequently introduced into the airways of naive animals they cause protracted lung and brain injury (FIGURE 10).

Hyperphosphorylated tau forms oligomers and larger aggregates that exhibit characteristics of amyloids (167, 169, 225, 287, 406–409). Antibodies recognizing amyloid oligomers detect tau in the supernatant after infection (26, 28, 29, 308). Moreover, antibodies that neutralize oligomeric forms of tau diminish and/or abolish transmissible cytotoxicity (26, 28, 29, 34, 308). Tau knockout endothelial cells do not express tau, do not generate tau oligomers after infection, and do not generate cytotoxic supernatant (26, 31). Tau is essential to generation of a stable, transmissible, and self-replicating endothelial cytotoxin in response to infection, especially after infection of *P. aeruginosa* strains/mutants that possess the type III secretion system and its effectors. Yet both cytotoxic tau and Aβ variants can be isolated together with the A11 antibody (34, 308). The nature of interaction between tau and Aβ after infection remains poorly understood.

### 4.2. Antimicrobial and Cytotoxic Aβ

**FIGURE 11.**
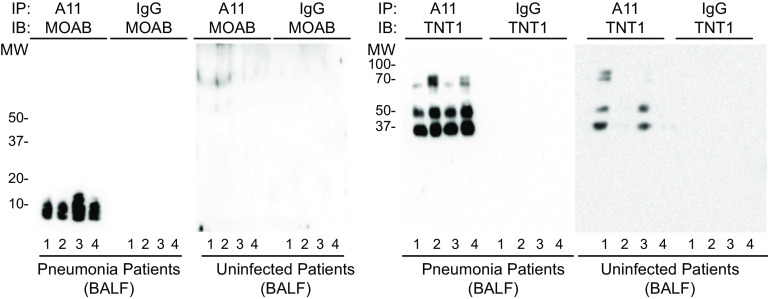
Cytotoxic tau and Aβ variants are present in the bronchoalveolar lavage fluid (BALF) of mechanically ventilated patients with ongoing pneumonia. BALF was collected from mechanically ventilated patients, and amyloids were immunoprecipitated (IP) with the A11 antibody and then immunoblotted (IB) for either tau (TNT1 antibody) or Aβ (MOAB antibody). Whereas pneumonia patients possess cytotoxic variants of both tau and Aβ, BALF from uninfected patients exhibited either minor or no immunoreactivity for tau and Aβ. MW, molecular weight. Adapted from Ref. 308, with permission from *American Journal of Respiratory and Critical Care Medicine*.

Much like the cytotoxic variants of tau, infection elicits cytotoxic Aβ production (FIGURE 11) (27). Aβ variants can be cytotoxic to microorganisms and to the host, as discussed in sect. 3.5. Considerable work has been dedicated to understanding the pathophysiological role of Aβ in neurodegenerative diseases, as in the amyloid hypothesis of Alzheimer’s disease.

More recently, investigators have resolved its role as an antimicrobial peptide (209, 376, 410–414). Antimicrobial peptides, including cathelicidins and LL-37, have long been studied as potential adjunctive treatments for microbial infection (415–418). Considering the multidrug resistance crisis, through which bacteria are rapidly evolving antibiotic resistance at a precipitous rate, interest in antimicrobial peptide research has gained notable momentum (417, 419–422). Many antimicrobial peptides, or host defense peptides, are derived from epithelial or canonical immune cells and are rich in α-helices, whereas mammalian β-defensin antimicrobial peptides possess a β-sheet architecture. Antimicrobial peptides often function bactericidally to disrupt the bacterial membrane or bacteriostatically via agglutination (416, 418, 423–426).

Kumar et al. (376) and Spitzer and colleagues (414) independently reported in 2016 that Aβ agglutinated bacteria in an antimicrobial peptide-like manner. Aβ_42_ oligomers adhered and nucleated amyloid fibril formation from the exterior of bacteria and yeast to aggregate offending pathogens (376, 414). In recent years, notable attention has been given to the similarities between amyloids and antimicrobial peptides, including their mechanism of supramolecular assembly (183, 413, 414, 427–429). Amyloids are an ancient means of defense and persistence and are expressed throughout all domains of life; they function in signaling, immunity, protection, adherence, and interspecies communication (199, 218, 412, 413, 427–440). Some mammalian amyloids implicated in host defense include Aβ (183, 376, 410, 412–414), α-synuclein (441–443), tau (444, 445), and s100 proteins (446–449).

Antimicrobial amyloids are released from pulmonary capillary endothelial cells constitutively, and cytotoxic Aβ variants are released in response to infection (29, 34, 450–452). *Pseudomonas aeruginosa* is one of the most commonly diagnosed organisms of hospital-acquired bacterial pneumonia (453, 454). Acute lung infection with *P. aeruginosa* induces the release of both cytoprotective amyloid antimicrobials (34) and cytotoxic amyloid pathogens (26, 28, 29, 31, 34, 308, 455). In response to *P. aeruginosa* infection, the functional phenotype of the endothelial amyloid produced (e.g., antimicrobial or cytotoxic) is dependent on whether the strain has a functional type III secretion system and which of the exoenzymes are injected into the host cell, particularly ExoY.

Infection with avirulent type III secretion system-deficient strains of *P. aeruginosa* elicits the release of heat-stable amyloid antimicrobial peptides from microvascular endothelium, whereas endothelial intoxication with type III secretion system effectors, particularly ExoU and ExoY, potentiates the release of endothelium-derived dysregulated tau oligomers that appear to facilitate the conversion of endothelial amyloid antimicrobials into a virulent, self-propagating amyloid-tau species (34). Although Aβ looks to be a significant contributor to endothelial amyloid antimicrobicity, other unresolved oligomeric amyloid species also play a role. *P. aeruginosa* T3SS-mediated injection of toxins ExoU/T (i.e., infection with laboratory strain PA103) into capillary endothelium produces only a partial reduction in the antimicrobial efficacy of endothelium-derived amyloids and a moderately virulent tauopathy. Endothelial intoxication with an ExoS/T/Y-competent clinical strain significantly attenuates antimicrobicity and promotes a highly virulent tauopathy. Injection of ExoY alone into capillary endothelium (i.e., laboratory strain that expresses only ExoY) is sufficient to ablate the antimicrobial activity of endothelial amyloid innate effectors and generate a virulent tauopathy commensurate with the ExoS/T/Y-competent clinical strain. Moreover, the immunodepletion of oligomeric tau rescues endothelial innate amyloid antimicrobicity and abrogates cytotoxicity, lending credence to the notion that dysregulated tau release drives the cytoprotective-to-cytotoxic conversion of endothelial amyloid species (34).

The type III secretion system effector ExoY also impacts the intracellular innate immune response. ExoY suppresses both TAK1-mediated innate immunity and antimicrobial endothelial amyloid release, suggesting a potential role for toll-like receptor signaling in the production and release of endothelial amyloids. Toll-like receptors are one of many pattern recognition receptors that detect a particular component of an infecting agent and induce innate immune signaling via activation of transcription factors NF-κB and activator protein-1 (AP-1) (456, 457). These transcription factors are often requisite to instigation of cytokine and antimicrobial peptide release (456–461). Indeed, toll-like receptor-2 has been reported to respond to Aβ as well as playing a role in its production and release (462–464). Focusing future studies on delineation of the signaling pathway and potential toll-like receptor mechanism responsible for endothelial amyloid production, release, and potentiation will be a crucial step forward in defining the role of endothelial amyloids, including Aβ, in innate defense of the alveolar-capillary barrier.

## 5. AMYLOID DISSEMINATION DURING INFECTION

### 5.1. Cytotoxic Tau and Aβ in Biological Fluids

Cytotoxic variants of tau and Aβ are produced by lung endothelium in vitro and by the lung in vivo, and they have been recovered from biological fluids in humans with infection (FIGURE 11). Evidence in support of this assertion relies on use of antibodies to detect, select for, and/or neutralize cytotoxic tau and Aβ and on animals with gene deletions, particularly tau knockout mice, as discussed below. Because amyloids occur in nature in diverse forms, parallel studies have been performed to determine the relative cytotoxicity of amyloids isolated from biological fluids. It is notable that to date cytotoxic variants of tau and Aβ have only been documented among patients with ongoing, or a recent history of, infection. Inflammation that accompanies critical illness in the absence of infection has not yet been shown to elicit cytotoxic tau and Aβ production. At present, only small clinical studies have been completed, and there is a significant need for a comprehensive multisite trial to further examine the link between infection, production of cytotoxic tau and Aβ variants, and end-organ dysfunction.

### 5.2. Bronchoalveolar Lavage Fluid

Cytotoxic variants of tau and Aβ have been recovered from the bronchoalveolar lavage fluid of humans and animals with pneumonia (28, 29, 308). Bronchoalveolar lavage fluid can be obtained from sputum culture, tracheal aspirates, and fiber-optic bronchoscopy. Importantly, sputum culture is only applicable to spontaneously breathing patients, and this approach collects very little fluid from the distal airways, i.e., it does not sample the lower respiratory tract. Tracheal aspirates require the placement of a flexible catheter into the endotracheal tube of a mechanically ventilated patient and blindly inserting and suctioning fluid from the distal airways. Fiber-optic bronchoscopy involves direct visualization with a bronchoscope, application of fluid into a wedged position, and collection of fluid from the distal airways.

Two of these methods can be used to collect lavage fluid from mechanically ventilated patients, each with a different advantage: there is better regional specificity with bronchoscopy and potential benefits of generality in tracheal aspirates. Cytotoxic variants on tau and Aβ within the distal airways have been detected with both tracheal aspirate and fiber-optic bronchoscopy approaches (28, 308).

New guidelines demonstrate a correlation between clinical symptoms and bronchoalveolar lavage fluid colony-forming units (dependent on method of collection), important to determine whether a patient has bacterial pneumonia or is merely colonized with bacteria (465). This is especially important, as acute infection worsens intensive care unit outcomes and it can lead to post-intensive care unit syndrome, with distal end-organ injury (466–471). Screening bronchoalveolar lavage fluid of mechanically ventilated patients with and without documented evidence for bacterial pneumonia, based on both clinical evidence and bronchoalveolar lavage fluid colony-forming units, revealed that cytotoxic tau and Aβ_42_ concentrations within the fluid portended end-organ dysfunction (Renema P, Pittet J-F, Brandon AP, Leal Jr SM, Gu S, Promer G, Hackney A, Braswell P, Pickering A, Rafield G, Voth S, Balczon R, Lin MT, Morrow KA, Bell J, Audia JP, Alvarez D, Stevens T, Wagener BM, unpublished observations). More specifically, an elevation in cytotoxic tau was associated with mortality, whereas an elevation in Aβ_42_ was associated with survival. Both tau and Aβ_42_ were positively associated with the four-point lung injury scale, use of vasopressors, and coagulopathy, indicating that elevations in these amyloids corresponded with worse lung injury, hemodynamic instability, and dysfunctional blood clotting.

Sampling bronchoalveolar lavage fluid for cytotoxic tau and Aβ may therefore provide important clinical information about susceptibility to end-organ injury while providing mechanistic insight into a cause of end-organ injury.

### 5.3. Blood

Cytotoxic tau and Aβ variants are present in the blood of patients with pneumonia (26). Blood is easily collected, especially from patients in the intensive care unit setting, via venipuncture, a central venous catheter, and/or an arterial line. Blood comprises a large fraction of red blood cells, a variable amount of white blood cells (depending on the severity, timing, and cause of infection), and fluid that contains nonadherent proteins, i.e., plasma/serum. Blood centrifugation enables separation of plasma/serum from blood cells. Studies to date have revealed elevations in tau and Aβ within the plasma fraction of the blood in blood and extracorporeal membrane oxygenators of patients and/or animals with pneumonia (26).

**FIGURE 12.**
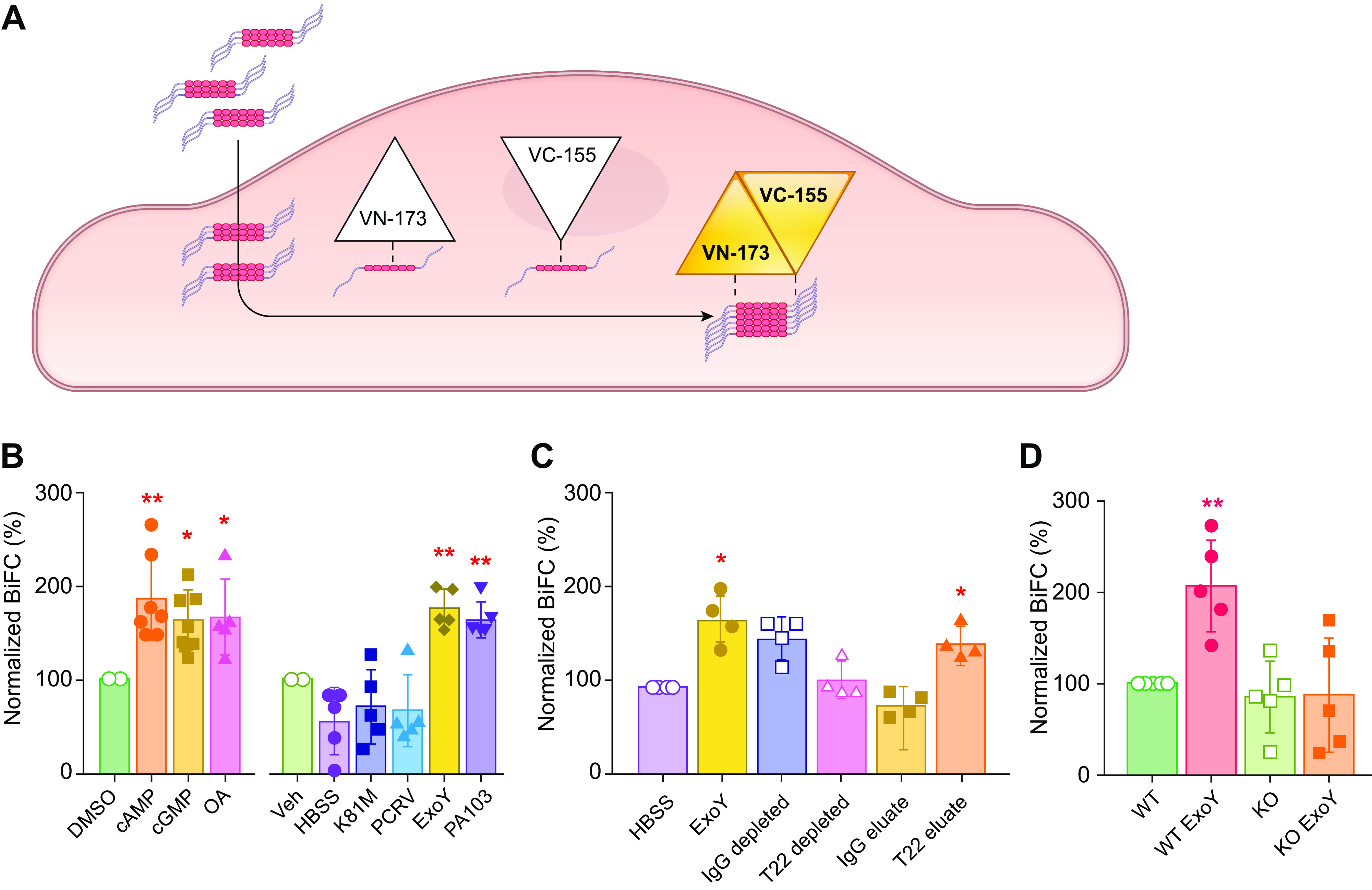
Infection-elicited cytotoxic tau promotes neuronal tau seeding. *A*: cytotoxic tau isolated from lung endothelium, plasma, and the cerebrospinal fluid and/or brain parenchyma after infection leads to tau seeding. The tau seeding assay utilizes bimolecular fluorescence to assess tau aggregation. The amino-terminal sequence (VN-173) and the carboxy-terminal sequence (VC-155) are each expressed as a fusion protein with monomeric tau. These individual sequences do not fluoresce, but in response to stimuli that lead to tau aggregation the amino- and carboxy-terminal fluorophores acquire fluorescence. *B*: biomolecular fluorescence (BiFC) is increased by positive controls, including cyclic nucleotides (cAMP and cGMP) and okadaic acid (OA). Supernatant from lung endothelial cells infected with type III secretion system-competent *Pseudomonas aeruginosa* strains, like PA103 and ExoY^+^ (PA103Δe*xoUexoT*::Tc pUCP*exoY*), but not from nonvirulent *P. aeruginosa* strains/mutants, promotes tau seeding. *C*: increased tau seeding is inhibited when cytotoxic tau variants are neutralized with the T22 antibody. *D*: brain homogenates from wild-type (WT) animals after infection cause tau aggregation, yet brain homogenates from uninfected animals and from infected tau knockout (KO) animals do not cause tau seeding. Statistical comparison was performed using ANOVA and Dunnett’s post hoc tests. Statistical significance versus (B) DMSO (B), Veh (C), or HBSS controls (D): *P < 0.05; **P < 0.005. Adapted from Ref. 31, with permission from *Journal of Biological Chemistry*. Figure created with BioRender.com, with permission.

The cytotoxic tau and Aβ isolated from the blood after infection injures cells in vitro and organs in vivo (26). More specifically, introduction of cytotoxic tau and Aβ isolated from the circulations of humans and animals into either the airways or blood of naive, i.e., otherwise uninfected, animals leads to lung and brain injuries. These amyloids seed neuronal tau (FIGURE 12) (26, 31). This seeding phenomenon was illustrated in two separate studies. First, whereas cytotoxic tau and Aβ were sufficient to impair long-term potentiation in wild-type mice, the brain was protected from injury in tau knockout mice. Moreover, infection did not elicit production of cytotoxic amyloid variants in the tau knockout mouse. Second, cytotoxic tau and Aβ nucleate, or “seed,” neuronal tau in an in vitro seeding assay, as described by Diamond and colleagues (472–475). In this assay, application of cytotoxic tau variants to cells promotes the aggregation of intracellular tau monomers, as assessed by either fluorescence resonance energy transfer or bimolecular fluorescence complementation. Plasma from patients 1, 7, and 14 days after pneumonia diagnosis drives tau aggregation in these assays, indicating that circulating tau variants are cytotoxic (Lin MT, unpublished observations). In contrast, plasma from uninfected control patients and uninfected mechanically ventilated patients is without effect. Evidence that cytotoxic tau variants are present in the circulation at least 14 days after infection is notable, since they remain present even after successful antibiotic therapy. This circulating source of cytotoxic tau may contribute to end-organ dysfunction in the aftermath of infection.

### 5.4. Cerebrospinal Fluid

Cytotoxic variants of tau and Aβ have been recovered from the cerebrospinal fluid by ∼48 h after lower respiratory tract infection (308). Cerebrospinal fluid is made via the ependymal cells of the choroid plexus. Cerebrospinal fluid clears waste products from the brain, controls acid-base balance within the central nervous system, and cushions and supports the central nervous system (476). The cerebrospinal fluid circulates through the lateral, third, and fourth ventricles as well as around the spinal cord. The roughly 150 mL of cerebrospinal fluid within the average human is turned over approximately four times per day (476). The cerebrospinal fluid contains protein, glucose, and limited cells, and sampling the cerebrospinal fluid for alterations in normal concentrations and for the presence of infection has diagnostic value, especially in critically ill patients.

Cerebrospinal fluid in critically ill patients is collected via either lumbar puncture or an extraventricular drain (308, 477). Lumbar puncture is performed in patients with a small spinal needle (used in subarachnoid blocks for parturient women) that reduces the risk of epidural hematoma and/or postdural puncture headache. However, performing lumbar puncture in mechanically ventilated, critically ill patients is more tenuous, given that many of them have coagulopathy and/or thrombocytopenia for various reasons, may be on prophylactic or therapeutic anticoagulation, and are sedated, leaving them unable to report neuropathy during the procedure (478). Cerebrospinal fluid can also be collected via an extraventricular drain. An extraventricular drain is a catheter that is placed (typically by neurosurgeons) in the lateral ventricle to drain cerebrospinal fluid and relieve intracranial pressure (479). The cerebrospinal fluid can be collected for molecular studies. Extraventricular drains are placed for clinical need only, limiting their widespread use for translational studies.

In a small study, cerebrospinal fluid was obtained via extraventricular drain in mechanically ventilated patients, with and without pneumonia (308). The fluid obtained from infected patients possessed amyloids that were cytotoxic to the brain (29, 308). Application of the fluid collected from uninfected mechanically ventilated patients to rodent hippocampi had no inhibitory effect on long-term potentiation. In stark contrast, however, the fluid collected from infected patients acutely abolished long-term potentiation. Neutralizing amyloids from the cerebrospinal fluid of infected patients normalized long-term potentiation. Moreover, eluting amyloids from the neutralizing antibodies and applying them in artificial cerebrospinal fluid to the rodent hippocampi also abolished long-term potentiation. This deleterious effect on long-term potentiation was seen after the cerebrospinal fluid was boiled, indicating that the injurious amyloids are heat stable. Cytokines were measured in the cerebrospinal fluid, and their concentrations did not change after amyloid neutralization. Moreover, cytokines would not withstand boiling. Thus, pneumonia initiates production of cytotoxic tau and Aβ variants that appear within the cerebrospinal fluid after infection; these variants impair neuronal information processing, and over time they lead to a reduction in the hippocampal spine density and impaired learning and memory in rodents (29, 455).

### 5.5. Other Biological Fluids

Cytotoxic variants of tau and Aβ have been recovered from the bronchoalveolar lavage fluid, blood, and cerebrospinal fluid of patients and animal subjects with ongoing pneumonia, yet other biological fluids relevant to dissemination of the variants have not been tested. For example, whether tau and Aβ are cleared through the gastrointestinal tract and/or renal filtration has not been tested, although urine is easily sampled and may provide insight into peripheral distribution and turnover. Saliva may be a sensitive sample of airway infection. Pleural fluid has not been tested, but ongoing work will assess whether this is a reservoir for cytotoxic tau and Aβ in pneumonia or in other causes of pleuritis.

### 5.6. Infection-Induced Amyloids: Coagulation and Myocardial Infarction

Clinical studies have reported a significant association between bacterial pneumonia and the later development of coagulation abnormalities in mechanically ventilated intensive care unit patients (480). The coagulation cascade is activated during infection by bacteria and viruses and corresponds to a mechanism of host defense against the spreading of the infection. In bacterial lung infection, the coagulation cascade is locally activated by the tissue factor expressed on lung epithelial cells that causes the release of fibrin within the lungs (481). Indeed, inhibition of the formation of thrombin and the depletion of fibrin have been associated with bacterial dissemination and increased mortality in pneumonia caused by *Klebsiella pneumoniae* (482). These results are supported by the fact that bacteria activate fibrinolysis, causing an increase in fibrin degradation and clot lysis that causes propagation of the bacterial infection (483). Thus, the activation of the coagulation extrinsic pathway causes the production of thrombin and stimulates platelet-neutrophil interactions that play an important role against the spreading of bacterial infection in the lung (483). Interestingly, the severity of the procoagulant activity associated with bacterial pneumonia also depends on the pathogen causing the pneumonia. For example, pneumonia caused by *Pseudomonas aeruginosa* is associated with an increase in the expression of the procoagulant activity and an increase in PAI-1 within the lung that may become deleterious for the host (484–486).

**FIGURE 13.**
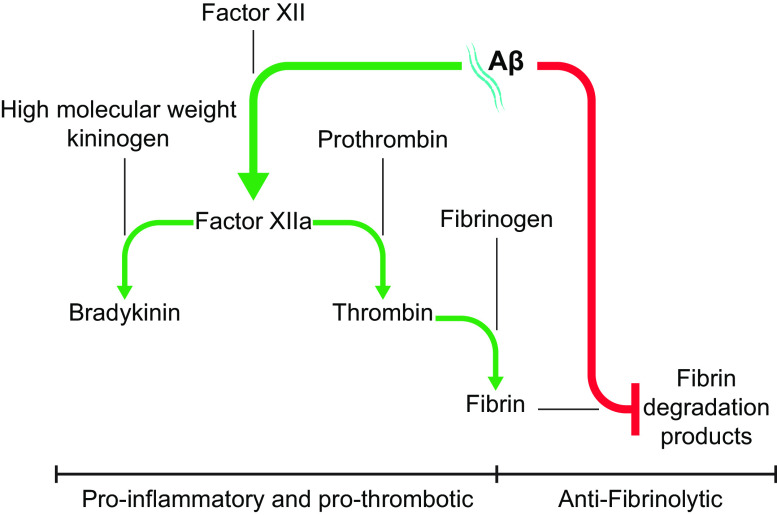
Beta-amyloid in the circulation can be proinflammatory, prothrombotic, and antifibrinolytic. Beta-amyloid interacts with factor XII, leading to production of the proinflammatory product bradykinin and generation of the prothrombotic product fibrin (illustrated in green). Beta-amyloid interacts with fibrinogen, which impairs fibrinolysis and reduces fibrin interaction with plasminogen, resulting in a profibrotic, antifibrinolytic environment (highlighted in red). Adapted from Ref. 497, with permission from *Current Opinion in Hematology*. Figure created with BioRender.com, with permission.

When the bacterial infection reaches the blood, it causes the development of sepsis with a systemic activation of immune and coagulation cascade called immunothrombosis that causes the development of end-organ injury. Importantly, under these circumstances, there is a constant cross talk between the aberrant immune cell activity and the dysregulated overactivation of the tightly controlled coagulation cascade that overcomes the benefit of limiting the spreading of the infection by a procoagulant milieu, thus causing microvascular thrombosis, tissue hypoxia, and death (487, 488). For example, epidemiological studies have shown that respiratory tract infections are associated with increased risk of vascular disease including arterial thrombosis (myocardial infarction and stroke) (489–495) as well as venous thromboembolism (496). Amyloid species are released by the lung endothelium into the blood and the lung distal air space of humans and rodents secondary to bacterial lung infection with bacterial pathogens, and these endothelial amyloids may contribute to hypercoagulation (FIGURE 13). The endothelial cytotoxic amyloids cause lung injury, cardiac dysfunction, and neuronal tauopathy (26, 31, 34, 308).

Amyloid species have been reported to cause coagulation abnormalities by several mechanisms (FIGURE 13). First, Aβ has been shown to cause impairment of angiogenesis, thus affecting the physiological ratio between ADAMTS13 and von Willebrand factor activity on endothelial cells and contributing to endothelial cell dysfunction (498–500). For example, infection of microvascular endothelial cells by ExoU-producing *Pseudomonas aeruginosa,* a bacterium that causes the release of cytotoxic Aβ by lung endothelial cells, induces the release of von Willebrand factor from cytoplasmic stores of these cells and increases the permeability of the lung endothelial barrier (501). Second, the contact coagulation system can be activated by Aβ, which in turn activates coagulation factor XII. The activation of factor XII has two major effects: it leads to the production of thrombin in the plasma that causes inflammation and thrombosis and activates kallikrein that also causes inflammation and increases endothelial permeability (502, 503). Interestingly, the knockout of factor XII in mice does not affect normal hemostasis. However, inhibition of the contact system of coagulation prevents the activation of factor XII by Aβ and protects mice from clot formation secondary to cerebral ischemia (504). Third, there are important interactions between coagulation factor XIII and Aβ peptides. Under physiological conditions, factor XIII plays a critical role in forming a stable fibrin clot. However, it has recently been shown that activated factor XIII can also cross-link Aβ into oligomers and to fibrin, thus increasing the deposition of Aβ oligomers in vivo (505). Fourth, Aβ has been shown to bind to fibrinogen and fibrin, causing the formation of abnormal blood clots that are more difficult to degrade by the fibrinolytic system (506–508). These abnormal fibrin clots that are difficult to be lysed by the fibrinolytic pathway may cause ischemia secondary to vascular thrombosis and the development of a chronic inflammatory state and cellular damage (509). Fifth, Aβ directly affects anticoagulant proteins by causing a decreased expression and shedding of the endothelial cell protein C receptor, thus decreasing the activation of protein C by its receptor (510). This result is of importance, as activated protein C has been shown to reduce in vitro cell release of Aβ and to significantly attenuate lung endothelial injury caused by lung infection with *P. aeruginosa* (511). Interestingly, the protection of the lung endothelium is largely provided by the cytoprotective, but not the anticoagulant, property of protein C (511).

These data indicate a complex relationship between cytotoxic amyloids and the vascular and hemostatic systems. Although activation of the coagulation cascade by antimicrobial amyloids may protect against dissemination of the infection, systemic activation of the coagulation cascade by these cytotoxic amyloids is deleterious and is part of the development of immunothrombosis associated with systemic infection.

As indicated, clinical studies have found that pneumonia substantially increases the risk for myocardial infarction, although the mechanisms responsible for this observation are unknown (489–495). Findings in pretranslational studies support the clinical observations (512). More specifically, myocardial ischemia-reperfusion injury is increased ∼30% 48 h after infection. Interestingly, the Aβ signaling system has been incriminated in this increase in myocardial infarction risk but not in the baseline ischemia-reperfusion injury. Gamma secretase activating protein knockout rats exhibit a similar degree of ischemia-reperfusion injury under baseline conditions, but the increase in ischemic zone area is abolished in the postinfection period. Future studies are needed to determine whether the postinfection myocardial ischemia-reperfusion injury is due to Aβ or other signaling roles of gamma secretase activating protein.

### 5.7. Infection-Induced Tau and Amyloids: a Lung-Brain Amyloid Axis That Impairs Learning and Memory

The brain is a highly specialized organ. It coordinates executive functions, like thought, planning, and learning. It facilitates homeostasis by processing afferent input information from all tissues and relaying responses via efferent neuronal pathways. It is responsible for unconscious functions, establishing the backdrop of emotions and urges while maintaining breathing, heart rate, and blood pressure. The brain accesses peripheral tissues via nerves emanating from the central nervous system, yet physically the brain is largely isolated from the chemical environment within the blood because of the restrictive nature of the blood-brain barrier and its associated cerebrospinal fluid barrier (500, 513). Because of the presence of these barriers and the relative absence of an evident lymphatic system, the central nervous system has long been referred to as being “immune privileged.” This terminology and concept is shifting to the idea that it is “immune specialized” with the discovery that the brain has dural lymphatic vessels (514, 515) and that the cerebrospinal fluid has direct access to skull bone marrow connecting the brain and its own unique immune system (516–518) and with the recent discovery of a fourth meningeal layer that compartmentalizes the subarachnoid space, termed the subarachnoid lymphatic-like membrane (519). Despite this restrictive barrier, some pathogenic stimuli, including amyloids, prions, microbes, and viruses, gain access to the brain. Although it is clear that amyloids emanating from sites that are peripheral to the brain can access the cerebrospinal fluid, important questions remain regarding how lung-derived amyloids, for example, may translocate from the lung to the brain (see sect. 5.7.1).

The general idea that amyloids emanating from a peripheral site can access the brain and cause disease came from recognition that certain misfolded proteins are “infectious.” Stanley Prusiner (520) identified a protein that possesses this property and coined the term “prion,” for “proteinaceous infectious particle.” The initially described prion diseases included kuru, transmissible spongiform encephalopathies, scrapie, and Creutzfeldt–Jakob disease, among others (521–526). In each of these cases, a naturally occurring prion protein, PrP^C^, misfolds to acquire a heat-stable, protease-resistant, transmissible, and cytotoxic form, the so-called PrP^SC^. Access of PrP^SC^ from one individual to the gut and/or blood of another naive human or animal recipient enables PrP^SC^ to access the brain: an amyloid peripheral to the brain translocates into the brain and causes a disease. Thus, these studies revealed the foundational principle that certain proteins, like PrP^C^, can acquire a dysfunctional conformation that is stable and is transmissible among humans/animals, resulting in neurological disease (527).

Whereas the principle of prion disease was first identified with PrP^SC^, the spongiform encephalopathies are rare degenerative brain disorders. Yet more recently, it has become apparent that multiple different proteins can acquire prion-like characteristics. These proteins are amyloidogenic (see sects. 3.4. and 3.5), and in some cases they are neurotropic in nature. In particular, amyloids that cause neurological diseases, such as tau and Aβ, can become heat stable, protease resistant, transmissible, and neurotropic to discrete brain regions. For example, amyloid variants isolated from the brains of diseased Alzheimer’s disease patients and injected into the brains of naive animals result in progressive neurodegenerative disease along the Alzheimer’s disease spectrum, in the same brain region of their origin (297). This example illustrates that brain-derived cytotoxic tau and amyloids cause disease in otherwise normal subjects. But, in addition, amyloids isolated from humans and/or animals that are injected into either the peritoneal cavity or the systemic circulation possess a neurotropic property (528–530). Significantly, these exogenous amyloid species gain access to the brain, where they also accumulate, propagate, and seed in the same brain region from which they originated, damaging the same type of neurons most susceptible to the amyloid species and recreating clinically relevant neurological deficits (531).

Although the proteinopathies described above are transmissible among subjects, a complete understanding of the mechanisms responsible for initiating protein misfolding is oftentimes lacking. Prion disease can be triggered by genetic mutations (532). Alzheimer’s disease and related tauopathies and amyloid diseases can also be initiated by direct genetic mutations, i.e., PSEN1 and MAPT (533). Yet the direct genetic causes of these diseases represent only a minority of the cases. The majority (∼90%) of known cases are sporadic in nature, meaning that the initiating cause is unknown (534). In sporadic cases, APOE-ε4 is an associated risk factor (535). Whereas prion disease is transmissible in nature as a natural course of disease, other proteinopathies are transmissible in experimental settings but have not yet been shown to be transmissible as a natural course of disease (219, 536).

**FIGURE 14.**
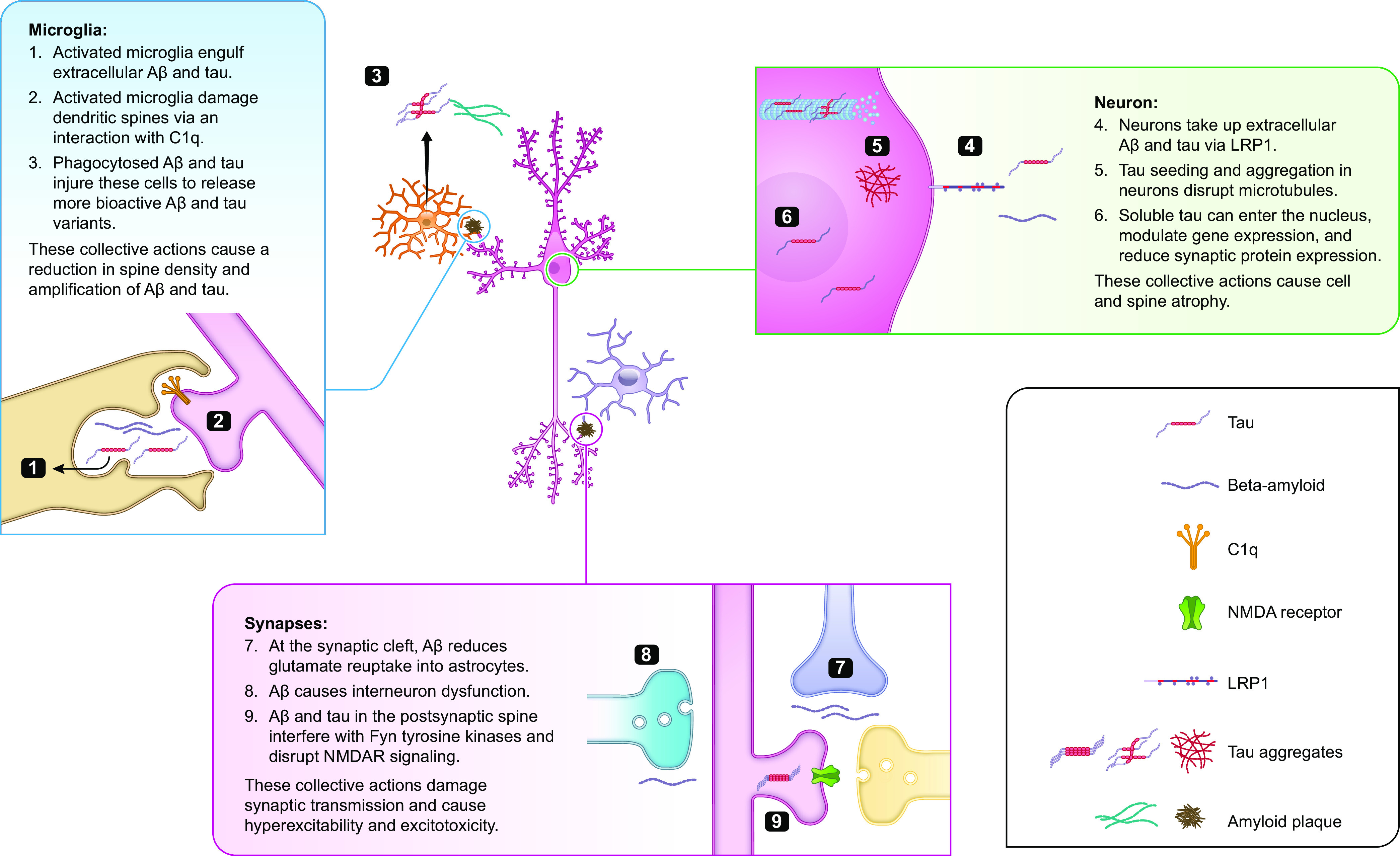
Cytotoxic tau and Aβ variants generated in the lung during infection access the cerebrospinal fluid and brain parenchyma, where they interact with neurons and cause hyperexcitability, impaired long-term potentiation (29, 308), and loss of hippocampal spines (455), all resulting in loss of learning and memory (29). LRP1, low-density lipoprotein receptor-related protein 1. Figure created with BioRender.com, with permission.

Infection is an incident cause of proteinopathy, leading to production of cytotoxic tau and Aβ variants that can injure the brain (FIGURE 14) (26–29, 31, 34, 308). Indeed, infection and critical illness are important causes of cognitive dysfunction (20, 537–541). Cognitive dysfunction can include anxiety, depression, and posttraumatic stress disorder, among other presentations. Notably though, it can promote mild cognitive impairment, an incident form of dementia within the Alzheimer’s disease spectrum (541). The mechanism of this phenomenon during the natural course of disease is unknown, although cytotoxic tau and Aβ may contribute. Heparan sulfate fragments may also contribute to cognitive dysfunction during sepsis (542, 543). These fragments are cleaved from the surface of endothelium and access the cerebrospinal fluid and brain, where they act to impair hippocampal long-term potentiation. It is noteworthy that heparan sulfate interacts with tau important for its cellular uptake (322, 323) and with Aβ (544, 545). Future studies are needed to address whether heparan sulfate fragments interact with cytotoxic tau and Aβ during infection, important to promote neurocognitive dysfunction. Whereas cytotoxic tau, cytotoxic Aβ, and heparan sulfates represent important infection-elicited mechanisms of neurocognitive dysfunction, improved quality of critical care limits the severity of delirium in the intensive care unit and therefore dementia in survivors (546–549).

**FIGURE 15.**
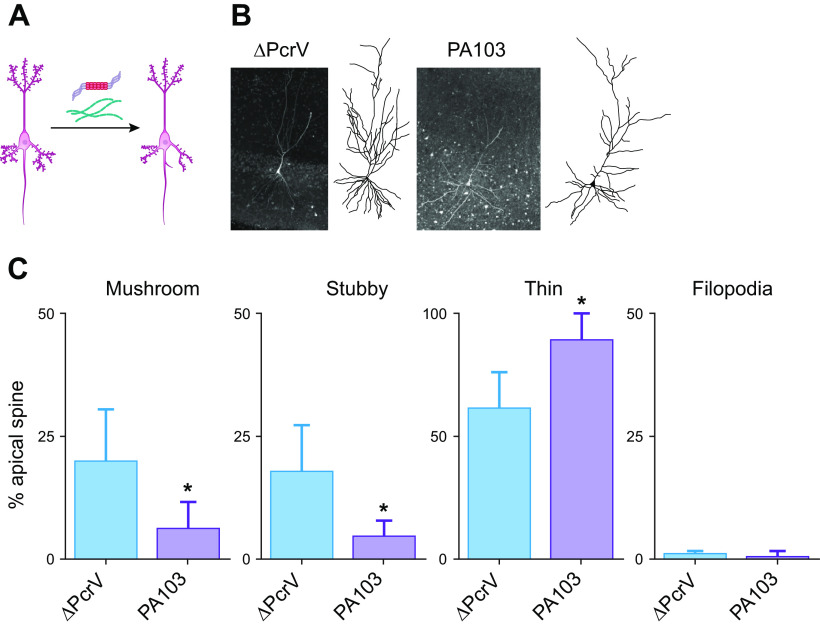
Infection-elicited cytotoxic tau and Aβ variants cause loss of hippocampal dendritic spines. *A*: schematic illustration of loss of spine density in the hippocampal neurons following exposure to cytotoxic tau and Aβ. *B*: hippocampal spine density was imaged and serial sections of Lucifer yellow-filled CA1 pyramidal cells were analyzed in animals infected with either type III secretion system-competent (i.e., PA103) or -deficient (i.e., ΔPcrV) *Pseudomonas aeruginosa* strains. PA103 infection caused a significant reduction in CA1 pyramidal cell spine density. *C*: analysis of the phenotype of spines lost after PA103 infection illustrating that mature apical spines were significantly reduced (i.e., mushroom and stubby) whereas the number of immature spines was increased (i.e., thin). *Different from ΔPcrV, *P* < 0.05, as analyzed by *t* test. Adapted from Ref. 455, with permission from *Scientific Reports*. Figure created with BioRender.com, with permission.

Infection-elicited cytotoxic tau and Aβ are long-lasting in biological fluids; they are heat stable, protease resistant, and transmissible (26–28, 34, 308). Studies resolving this phenomenon examined bacterial causes of nosocomial pneumonia, although some viruses have also been implicated. These cytotoxic tau and Aβ variants can be produced by lung capillary endothelium in vitro, and they are generated by the lung during the course of infection. They are found in the bronchoalveolar lavage fluid and blood during infection, and they last long after the infection has been effectively treated with antibiotics. They appear in the cerebrospinal fluid within 48 h of the onset of infection, and when they are in the brain they are sufficient to induce hyperexcitability, impair hippocampal long-term potentiation, and drive remodeling and ultimately loss of neuronal spines (FIGURE 14 AND FIGURE 15) (29, 308, 512). It is notable that a feedforward relationship exists between development of pneumonia and cognitive dysfunction: pneumonia can reduce cognitive function, and, in turn, worsening cognitive function increases the risk of subsequent bouts of pneumonia (550). With evidence that cytotoxic tau and Aβ variants are produced in the lung by infection, that they access the circulation and the cerebrospinal fluid, and that they disrupt neuronal information processing and decrease hippocampal spine density, it is important to determine how they translocate from the peripheral circulation into the brain as a cause of neurological deficits.

#### 5.7.1. Lung-to-brain amyloid entry across the blood-brain barrier.

The blood-brain barrier separates the blood from the brain while allowing for adequate gas and nutrient exchange. The blood-brain barrier is formed by endothelial cells and their intercellular tight junctions that limit paracellular flux (551–553). The blood-brain barrier is supported by brain pericytes and astrocytic endfeet. In contrast to leaky vessels in other systemic circulatory beds, the blood-brain barrier restricts the passage of molecules into and out of the central nervous system. For example, pathogens and neurotoxic blood-derived cells are not normally able to cross the blood-brain barrier to gain entry to the brain (513, 553). Meanwhile, active transport from the brain to the blood is ongoing, clearing waste products and other molecules (513, 553).

**FIGURE 16.**
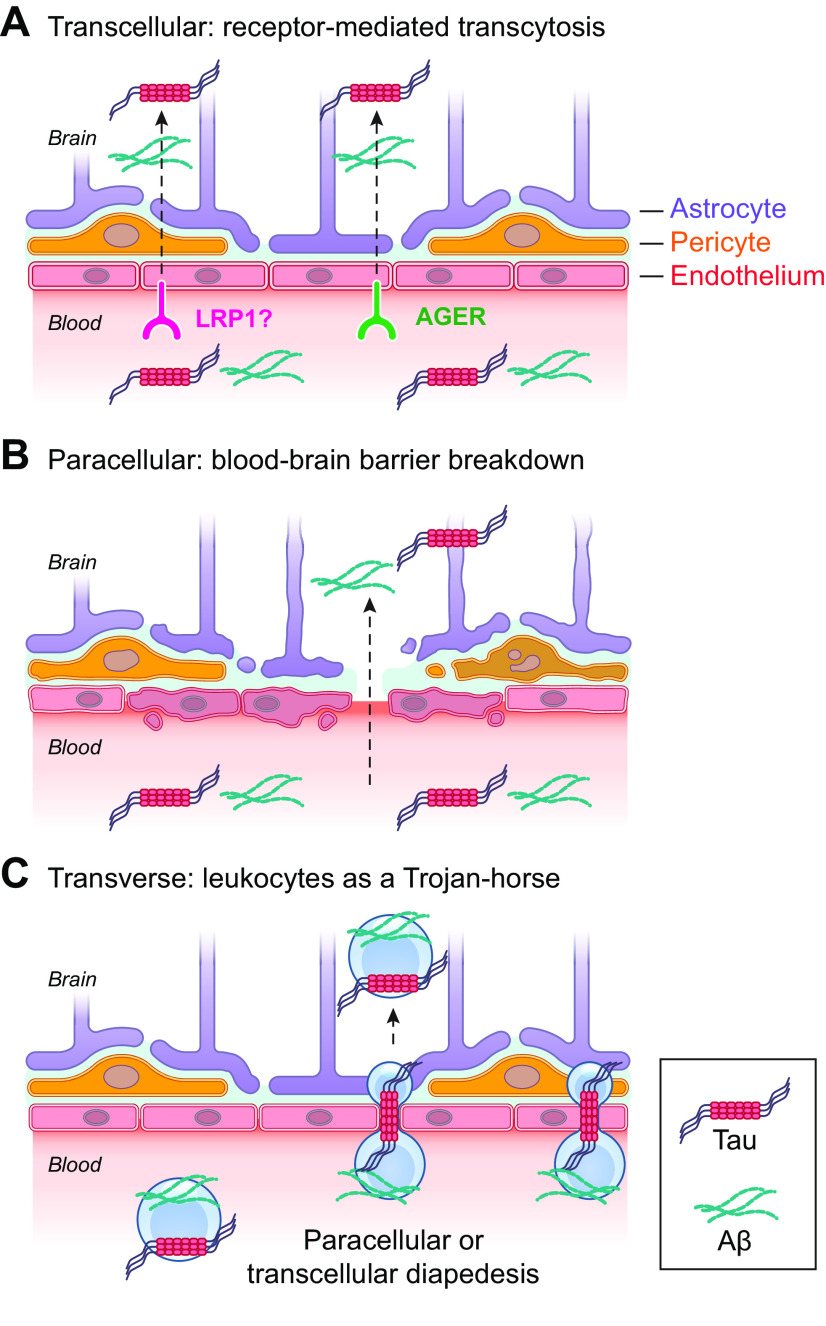
Routes of lung-to-brain amyloid entry across the blood-brain barrier. There are 3 possible pathways across the blood-brain barrier that Tau and Aβ may take, including transcellular (*A*), paracellular (*B*), and transverse (*C*) passageways. *A*: brain endothelial cells (pink) and their tight junctions form the blood-brain barrier, which is supported by pericytes (orange) and astrocyte endfeet (purple). Aβ has been demonstrated to cross the barrier from blood to brain by receptor-mediated transport via advanced glycosylation end product-specific receptor (AGER, formerly known as RAGE). Recent studies have implicated AGER in the transport of tau into microglia and neurons. Low-density lipoprotein receptor-related protein 1 (LRP1) is predominantly localized on the abluminal side of endothelial cells but is also present at the luminal side. LRP1 has been demonstrated to transport Aβ and tau. *B*: the blood-brain barrier breaks down in normal aging, infection, and hypoxia, allowing uncontrolled entry of molecules in the blood, such as tau and Aβ, to enter the brain. *C*: previous studies have shown that stimuli can use host cells, such as leukocytes, as a Trojan horse to cross the blood-brain barrier by paracellular or transcellular diapedesis.

For noxious stimuli to cross the blood-brain barrier and enter the brain, three pathways are possible, including *1*) transcellular *2*) paracellular, and *3*) transverse passageways (FIGURE 16). For example, the transcellular transport can be breached with an uncontrolled increase in passive or active transcytotic activity. Several blood-brain barrier peptide transport mechanisms exist, including receptor-mediated, adsorptive-mediated, carrier-mediated, and nonspecific passive diffusion (554). There are several specific receptor-mediated transporters expressed in brain endothelium under physiological or pathological conditions that have been implicated in amyloid transport across the blood-brain barrier (555, 556); these pathways are further described in sect. 5.7.3. The paracellular passage is guarded by endothelial tight junctions, and it can be disrupted after endothelial/epithelial injury and is often termed “breakdown” of the barrier. The transverse passage is a unique one, because in this case the stimuli “hijack” host cells, such as leukocytes, and use these cells as Trojan horses to bypass the barrier (i.e., hematogenous pathway) (323, 557–559). Although these passages can be described separately, they may be triggered by the same factors. For example, inflammatory factors have been implicated to play an important role in increased paracellular and transcellular permeability, thereby impairing neurocognitive function (119).

#### 5.7.2. Blood-brain barrier breakdown with aging, infection, hypoxia, and amyloids.

Studies using dynamic contrast-enhanced magnetic resonance imaging have reproducibly identified increased blood-brain barrier permeability in aged individuals (560, 561), which is worsened in individuals with mild cognitive impairment (560, 562–564). Systemic inflammation has been demonstrated to disrupt the blood-brain barrier in several studies, as recently reviewed (565). Lipopolysaccharide, viral RNA, systemic infection, inflammatory mediators, and cytokines reduce blood-brain barrier tight junctions and/or increase blood-brain barrier permeability (565). In addition, hypoxia and reoxygenation have been shown to trigger blood-brain barrier breakdown (551, 566, 567) through mitochondrial release of reactive oxygen species and/or by activating the hypoxia-induced factor-1 pathway (568, 569).

In Alzheimer’s disease, there is increased blood-brain barrier breakdown (119, 553, 570). Furthermore, *App*, *Psen1*, and *P301l* transgenic models with increased Aβ or tau production have vascular abnormalities and damage, as recently reviewed (552). Whether tau or Aβ produced in the periphery in response to infections, such as pneumonia, causes blood-brain barrier breakdown has yet to be determined (119).

#### 5.7.3. Blood-brain barrier transporters of tau and A**β**.

There is a significant body of research addressing transcellular, receptor-mediated bidirectional transport of Aβ across the blood-brain barrier (555, 556, 560). However, the exact mechanism of this transport remains unknown. Below we highlight key receptors involved in Aβ and perhaps tau transport across the blood-brain barrier.

##### 5.7.3.1. advanced glycosylation end product-specific receptor.

Advanced glycosylation end product-specific receptor (AGER, formerly RAGE) is a member of the immunoglobulin superfamily of cell surface molecules (571). It has one V-type domain responsible for ligand interactions, two C-type domains, and a 43-amino acid cytoplasmic tail essential for signaling (572, 573). *AGER* is normally expressed at relatively low levels in endothelial cells, pericytes, glia, and neurons in the brain and is also expressed in other cell types and organs in the body, including the lung (573). *AGER* is highly (i.e., predominantly) expressed in alveolar type I epithelial cells in the lung (99). AGER levels of protein are elevated in normal aging and disease states (574), which consequently increases inflammation and promotes oxidative stress (573). AGER is a multiligand receptor and can bind Aβ, advanced glycation end products, advanced oxidation protein products, amphoterin, S100B/calgranulins, nonenzymatically glycated protein or lipid products, and high-mobility group box 1 (555, 573).

Under pathological conditions, AGER is involved in the transport of circulating Aβ from blood to brain (575–577). AGER-Aβ interactions at the blood-brain barrier triggered NF-κB-dependent endothelial release of endothelin-1 and reduced cerebral blood flow (575). Investigations of AGER-tau interactions are limited. However, a recent study reported that AGER is involved in tau transmission in neurons and microglia, where it facilitates tau pathology progression and behavioral deficits (578). Other studies have linked increased levels of AGER in the brain to Aβ accumulation and tau phosphorylation in sepsis (579) and parasitosis (579–581). Furthermore, systemic AGER inhibition with circulating AGER antibodies reduced lipopolysaccharide-induced systemic inflammation (582). Future research should determine whether AGER is involved in the transport of tau across the blood-brain barrier and whether AGER inhibitors such as FPS-ZM1, already shown to be safe in clinical trials, are able to prevent neurocognitive decline associated with lung infection by reducing tau and Aβ brain entry and/or by reducing systemic inflammation.

##### 5.7.3.2. low-density lipoprotein receptor-related protein 1.

LRP1 is expressed at the blood-brain barrier by endothelial cells (both luminally and abluminally) and pericytes (513, 553, 555, 556, 559) and has been suggested to be a unidirectional transport system (583, 584). However, most studies have demonstrated LRP1 in the clearance of ligands, especially Aβ, across the blood-brain barrier from brain to blood (555, 556, 585, 586). LRP1 exists in two physiological forms: a cell surface-bound form (i.e., LRP1) and a truncated soluble form (i.e., sLRP1) (556). At the blood-brain barrier, LRP1 has two subunits, including a 500-kDa extracellular α-chain and a 100-amino acid intracellular β-chain, separated by a transmembrane domain and a short extracellular extension (558). More than 40 known ligands can bind LRP1 (587), including tau (323, 557) and Aβ (555, 556, 585, 586), and be endocytosed. The clearance of Aβ from the brain via LRP1 involves receptor-mediated transcytosis regulated by phosphatidylinositol binding clathrin assembly protein (588). No study to date that we could identify has investigated whether tau or Aβ could be transported via LRP1 from blood to brain.

#### 5.7.4. Lung-to-brain amyloid entry across the blood-cerebrospinal fluid barrier.

The blood-cerebrospinal fluid barrier is located at the choroid plexuses and is responsible for production of cerebrospinal fluid from the blood, and it is at the arachnoid membrane between the dura and the subarachnoid that returns cerebrospinal fluid into the blood circulation (584). The choroid plexuses are the major interface between the blood and cerebrospinal fluid because of the large vascular surface area. At the choroid plexuses, cerebromicrovascular endothelial cells are fenestrated and permeable, and the barrier is formed by the adjacent epithelial-like ependymal cells that line the cerebral ventricles (561). The ependymal cells express intercellular tight junctions close to the cerebrospinal fluid side of these cells, restricting paracellular transport. The blood-cerebrospinal fluid barrier is slightly more permeable than the blood-brain barrier. Specific transport proteins and transcytotic activity create cerebrospinal fluid and aqueous flow from blood.

Conversely, pathology such as sepsis increases the expression of amyloid transporters on the luminal side of endothelium and enhances transport of humoral amyloids into the brain (567). The luminal endothelial transporters may transport amyloid species, either extracted from diseased brains and injected into the blood or generated after lung infection, from the circulation into the brain (528, 589–591). Notably, the exogenously injected diseased brain amyloid species seed in the same brain region from which they originated, damaging the same type of neurons most susceptible to the amyloid species and recreating neurological deficits. It is currently unclear where lung-derived amyloids translocate to or in which brain regions in addition to the hippocampus these amyloids accumulate.

#### 5.7.5. Lung-to-brain entry via the nervous system.

Neuronal transport of lung-derived cytotoxic tau and Aβ variants from the airway to the brain has not been tested. However, at least two direct pathways enable lung pathogens to reach the central nervous system via neuronal transport. The first clue comes from coronavirus (CoV) studies. An early retrospective report from Wuhan, China, where the SARS-CoV-2 pandemic originated, indicated that 36% of 214 admitted patients exhibited signs of central nervous system abnormalities (592). Independent studies subsequently corroborated this observation and further indicated that up to 50% of SARS-CoV-2-positive patients demonstrated deficiencies in smell sensation (592, 593), incriminating nasal epithelium and olfaction in viral neural transmission. In the past two coronaviral outbreaks (SARS-CoV and MERS-CoV in 2002 and 2012, respectively), studies have demonstrated that CoV can reach the brain via the nasal cribriform plate-olfactory bulb. In rodents, St-Jean et al. (594) reported that 3 days after CoV nasal infection viral antigens are detectable in the olfactory bulb but not in perivascular blood cells. CoV could infect glial and neuronal cells (595). At 7 days after infection, viruses were found throughout the whole brain, leading to neural demyelination, acute encephalitis, and rapid death. Importantly, ablation of the olfactory bulb prevented viral spread into the central nervous system (596). These studies suggest that certain CoV strains are capable of invading the brain via the olfactory bulb. These viruses can replicate and propagate from the olfactory bulb to cells in the central nervous system.

The second direct pathway through which respiratory viruses reach the central nervous system was revealed from influenza virus studies. Influenza viruses cause seasonal flu, and associated neurological complications include seizures, encephalopathy, myelitis, and Guillain–Barré syndrome, among others. Stroke is also a known sequela, because some influenza viral strains disrupt the blood-brain barrier or the blood-cerebrospinal fluid barrier whereas others do not (these pathways are discussed below). Specifically, influenza virus can spread from the lungs to the central nervous system by a transneural route, via the vagus and trigeminal nerves. Studies using highly virulent strains of influenza virus inoculated into mice intranasally showed viral antigens in the vagal and trigeminal ganglia 3 days after infection (597–599), followed by detection in the brain at 7 days (600). Interestingly, whereas intranasal inoculation of influenza virus leads to bronchitis, viral detection in the mucosal epithelium of the airway, and nonsuppurative encephalitis, intravenous injection does not lead to respiratory or central nervous system pathology (599). In vitro studies showed that influenza virus in neuronal culture can utilize retrograde axonal transport (601). These studies indicate that certain strains of influenza viruses reach the central nervous system from the respiratory system-innervating vagus and trigeminal nerves via a retrograde transduction mechanism.

The CoV and influenza examples provide evidence for retrograde transport through neurons as a mechanism by which noxious stimuli access the brain. It is notable that loss of smell, similar to symptoms in SARS-CoV-2 infections, is a common early occurrence in neurodegenerative disorders (602). Exposure of the olfactory epithelium and the olfactory bulb to tau and Aβ has been considered a potential mechanism of disease propagation, although its role in the natural course of disease has not yet been confirmed. Nonetheless, experimental studies reveal that introduction of tau and Aβ aggregates into either the nasal passages or the tongue results in transmission to the brain (603). Since both cytotoxic tau and Aβ variants have been recovered from the bronchoalveolar lavage fluid of patients with infection, their access from the lung parenchyma to the airway must be considered. Recently, Bastarache and colleagues (604, 605) demonstrated that heat moisture exchange filter fluid can be collected from mechanically ventilated patients and, further, that the fluid contains soluble factors that reflect their concentrations in the bronchoalveolar lavage fluid. This finding indicates that factors produced in the distal airway can access the upper airway, where they may have contact with the olfactory epithelium and lingua. Future studies are warranted to assess whether retrograde neural transport represents a mechanism by which lung-derived cytotoxic tau and Aβ variants can access the brain.

## 6. CONCLUSIONS AND FUTURE DIRECTIONS

Lung capillary endothelium utilizes tau to stabilize microtubules, and it expresses amyloid precursor protein and the relevant secretases that mediate the nonamyloidogenic and amyloidogenic pathways, including production of Aβ (and potentially other amyloid precursor protein products) important for its antimicrobial properties. Lower respiratory tract infections drive production of cytotoxic tau and Aβ variants within the lung. Communication between the virulence arsenal of the microorganism and the host seems to be essential to production of cytotoxic variants, e.g., type III secretion system effectors. Once produced, these variants can be retrieved from the bronchoalveolar lavage fluid, the blood, and the cerebrospinal fluid of infected subjects. The cytotoxic variants exhibit properties of prion disease: they are heat-stable, transmissible, self-replicating cytotoxins, and they contribute to end-organ dysfunction in the aftermath of infection. It is notable that they can “seed” neuronal tau as a potential mechanism of disease propagation. Lung-derived cytotoxic tau and Aβ variants are products of interkingdom communication, and they are injurious to the host. Cytotoxic tau and Aβ variants represent a previously unrecognized target for medical therapy relevant to end-organ dysfunction.

Important questions remain. It will be important to resolve all of the cells within the lung parenchyma that are capable of producing cytotoxic tau and Aβ variants during infection and determine the relative contribution(s) of each cell type. More specifically, some populations of alveolar epithelial cells and alveolar pericytes appear to possess the molecular machinery to produce either tau or Aβ. Studies addressing the relative importance of each of these cell types to cytotoxic tau and Aβ production during the natural course of infection have not yet been completed.

At present, virulence mechanisms contributing to production of cytotoxic tau and Aβ are only partially resolved; in many cases, how microorganisms communicate with the endothelium to drive cytotoxin production is unknown and needs to be addressed. Structural studies are needed to assess the molecular basis of the tau and Aβ cytotoxic activity, to assess whether tau and Aβ interact after infection, and to determine whether they interact with other injurious molecules, like heparan sulfate. Whereas the presence of cytotoxic tau and Aβ in biological fluids is associated with infection, future studies are needed to assess their strength as biomarkers of infection and to determine whether they are sensitive indicators of infection even in the absence of positive cultures. To this extent, metagenomic studies will provide molecular insight regarding the linkage between infection and cytotoxic tau and Aβ. Mechanisms of cytotoxic tau and Aβ turnover have not been explored. Future studies addressing the half-life of the cytotoxins, their tissue-specific biodistribution, and their mechanism(s) of degradation and/or elimination are essential. It will be important to learn whether the host produces an antibody-mediated immune response targeting cytotoxic variants, or whether the cytotoxins can be cleared through proteosome-mediated degradation pathways, renal filtration, and/or gastrointestinal transport. Infection-elicited cytotoxic tau variants have been shown to “seed” neuronal tau, meaning they can cause neuronal tau aggregation as a feedforward mechanism of disease. The short-term and long-term consequences of this phenomenon need to be studied, and it will be important to assess whether a similar seeding mechanism occurs in peripheral tissues, like lung capillary endothelium. Since cytotoxic tau and Aβ contribute to end-organ dysfunction, clinical studies need to address whether targeting them during infection with neutralizing antibodies or other approaches improves patient outcomes. Pneumonia is a cause of cognitive dysfunction, and cognitive dysfunction increases susceptibility to pneumonia. Recurrent infections may contribute to chronic neurodegenerative disease(s), as hypothesized in the infectious hypothesis of Alzheimer’s disease. It is not clear whether the infection-elicited cytotoxic tau and Aβ variants share any structural homology with variants found in chronic neurodegenerative diseases, or whether they are entirely unique. Future studies addressing the relevance of recurrent infections as a trigger of chronic disease in susceptible individuals is warranted.

## GRANTS

This work was supported by the Department of Health and Human Services, including HL66299 (T.S., R.B.), HL148069 (T.S., R.B., J.-F.P.), HL140182 (M.T.L, T.S., R.B., J.-F.P.), AG058780 (A.R.N.), GM127584 (B.M.W.), GM127584-04S1 (B.M.W.), HL143017 (J.-F.P.), HL076125 (S.V.), 1F31HL147512-01 (S.V., T.S.), HL007778 (S.V.), and REAP220049 (S.V.). In addition, J.C.S. is supported by the German Research Foundation (SCHU 3147/4-1) and CORE100Pilot Advanced Clinician Scientist Program of Hannover Medical School funded by Else-Kröner-Fresenius Foundation (EKFS, 2020_EKSP.78) and the Ministry of Science and Culture of Lower Saxony.

## DISCLOSURES

No conflicts of interest, financial or otherwise, are declared by the authors.

## AUTHOR CONTRIBUTIONS

M.T.L., S.V., A.R.N., J.C.S., and T.S. prepared figures; R.B., M.T.L., S.V., A.R.N., B.M.W., J.-F.P., and T.S. drafted manuscript; R.B., M.T.L., S.V., A.R.N., J.C.S., B.M.W., J.-F.P., and T.S. edited and revised manuscript; R.B., M.T.L., S.V., A.R.N., J.C.S., B.M.W., J.-F.P., and T.S. approved final version of manuscript.
